# Adaptation of a microbial community to demand-oriented biological methanation

**DOI:** 10.1186/s13068-022-02207-w

**Published:** 2022-11-16

**Authors:** Hoda Khesali Aghtaei, Sebastian Püttker, Irena Maus, Robert Heyer, Liren Huang, Alexander Sczyrba, Udo Reichl, Dirk Benndorf

**Affiliations:** 1 Bioprocess Engineering, Max Planck Institute for Dynamics of Complex Technical Systems, Sandtorstraße 1, 39106 Magdeburg, Germany; 2 Bioprocess Engineering, Otto Von Guericke University Magdeburg, Universitätsplatz 2, 39106 Magdeburg, Germany; 3 Applied Biosciences and Process Engineering, Anhalt University of Applied Sciences, Bernburger Straße 55, Postfach 1458, 06366 Köthen, Germany; 4 Center for Biotechnology (CeBiTec), Genome Research of Industrial Microorganisms, Bielefeld University, Universitätsstraße 27, 33615 Bielefeld, Germany; 5 Institute for Bio- and Geosciences (IBG-5), Forschungszentrum Jülich GmbH, Wilhelm-Johnen-Straße, 52428 Jülich, Germany; 6 Database and Software Engineering Group, Otto Von Guericke University Magdeburg, Universitätsplatz 2, 39106 Magdeburg, Germany; 7 Faculty of Technology and Center for Biotechnology (CeBiTec), Bielefeld University, Universitätsstraße 25, 33615 Bielefeld, Germany; 8 Multidimensional Omics Analyses group, Leibniz-Institut für Analytische Wissenschaften – ISAS – e.V., Bunsen-Kirchhoff-Straße 11, 44139 Dortmund, Germany

**Keywords:** Renewable energy, Power to methane, Biological methanation, Biogas upgrade, Hydrogenotrophic methanogens, Acetoclastic methanogens, Hydrogen starvation, Metaproteomics, Microbial food web

## Abstract

**Background:**

Biological conversion of the surplus of renewable electricity and carbon dioxide (CO_2_) from biogas plants to biomethane (CH_4_) could support energy storage and strengthen the power grid. Biological methanation (BM) is linked closely to the activity of biogas-producing *Bacteria* and methanogenic *Archaea*. During reactor operations, the microbiome is often subject to various changes, e.g., substrate limitation or pH-shifts, whereby the microorganisms are challenged to adapt to the new conditions. In this study, various process parameters including pH value, CH_4_ production rate, conversion yields and final gas composition were monitored for a hydrogenotrophic-adapted microbial community cultivated in a laboratory-scale BM reactor. To investigate the robustness of the BM process regarding power oscillations, the biogas microbiome was exposed to five hydrogen (H_2_)-feeding regimes lasting several days.

**Results:**

Applying various “on–off” H_2_-feeding regimes, the CH_4_ production rate recovered quickly, demonstrating a significant resilience of the microbial community. Analyses of the taxonomic composition of the microbiome revealed a high abundance of the bacterial phyla *Firmicutes*, *Bacteroidota* and *Thermotogota* followed by hydrogenotrophic *Archaea* of the phylum *Methanobacteriota*. Homo-acetogenic and heterotrophic fermenting *Bacteria* formed a complex food web with methanogens. The abundance of the methanogenic *Archaea* roughly doubled during discontinuous H_2_-feeding, which was related mainly to an increase in acetoclastic *Methanothrix* species. Results also suggested that *Bacteria* feeding on methanogens could reduce overall CH_4_ production. On the other hand, using inactive biomass as a substrate could support the growth of methanogenic *Archaea*. During the BM process, the additional production of H_2_ by fermenting *Bacteria* seemed to support the maintenance of hydrogenotrophic methanogens at non-H_2_-feeding phases. Besides the elusive role of *Methanothrix* during the H_2_-feeding phases, acetate consumption and pH maintenance at the non-feeding phase can be assigned to this species.

**Conclusions:**

Taken together, the high adaptive potential of microbial communities contributes to the robustness of BM processes during discontinuous H_2_-feeding and supports the commercial use of BM processes for energy storage. Discontinuous feeding strategies could be used to enrich methanogenic *Archaea* during the establishment of a microbial community for BM. Both findings could contribute to design and improve BM processes from lab to pilot scale.

**Supplementary Information:**

The online version contains supplementary material available at 10.1186/s13068-022-02207-w.

## Background

Achieving a climate-neutral Europe by 2050 requires a significant increase in variable renewable electricity resources (VRERs), such as wind and photovoltaic farms. To maintain the stability of the power grid, VRERs’ intermittency and fluctuations have to be balanced [1]. In the “Gas for Climate” study from 2019, the European Biogas Association proposed an “Optimised Gas Scenario” to fully decarbonise Europe’s energy system at the lowest societal cost [2]. Compared to the proposed “Minimal Gas Scenario”, implementing this scenario will smartly integrate VRERs and renewable gases, which can save about 217 billion euros annually across the energy sector by 2050 [2].

As reported in the “Optimised Gas Scenario”, the application of biogas and biomethane can decrease about 10–13% of greenhouse gas emissions [2]. Biogenic CO_2_, from the combustion of biogas and biomethane, recirculates in a “short carbon cycle”, while fossil-based CO_2_ is released after millions of years of underground storage [2]. Biogas, as a sustainable alternative energy source, suffers from its low calorific value, which is caused by the presence of CO_2_ (30–50%) [3]. “First-generation of upgrading” techniques such as pressure swing adsorption or amine extraction only remove CO_2_ and convert biogas to pure biomethane [3, 4]. In contrast, “second generation of upgrading” techniques such as in/ex-situ methanation or syngas conversion into liquid organic molecules focus on using CO_2_ as a carbon source to store power produced from VRERs [4]. Therefore, implementing “second-generation upgrading” technology is necessary to reach climate neutrality policies [1, 4, 5]. An economic, flexible and efficient alternative could be a bridge between the gas and the power network in which the extra amount of CO_2_ in biogas and the surplus of renewable electricity (i.e., renewable H_2_) are converted to CH_4_ and water [1]. Such a “power to methane” technique will improve the efficiency of biogas production, help to reduce storage and fluctuations barriers of VRERs and support the progression toward climate neutrality [1].

Sustainability, mild conditions, high impurities tolerance and assumed dynamics (flexibility regarding load variations) of biological methanation (BM) processes are reasons that could outpace the Sabatier reaction as a method for thermochemical methanation [1, 6]. Despite the high potential of BM, its technical implementation is still restricted because of the insufficient exploration of its efficiency, stability and productivity [7]. Furthermore, a better characterisation of microbial community dynamics in BM processes can lead to a better understanding of the metabolic potential of BM [7, 8]. Recent progress in microbial community analyses, e.g., of full-scale biogas plants, provided valuable tools that could be applied to monitor microbial communities and process control [8]. Alteration in BM process parameters, e.g., fluctuation in H_2_-feeding, may cause variation in the microbial community and limit the system’s efficiency and productivity. Therefore, in-depth knowledge of the microbial adaptation to changes in the process conditions is crucial to the systematic scale-up of BM processes [7, 9].

To follow the intermittency of VRERs, flexibility as well as the robustness of energy storage technologies need to be considered [4]. While discontinuous H_2_-feeding can damage chemical methanation catalysts, BM processes handled by microbial methanogenic *Archaea* were demonstrated to be less sensitive to H_2_ oscillations [10]. Nevertheless, changes in H_2_-feeding could cause alterations in the composition of microbial communities and subsequent process disturbances result in a reduction in biomethane production yields. The flexibility of BM processes regarding process changes is due to the fact that complex microbial communities can adapt themselves relatively fast to environmental upsets [11]. Interactions between the members of microbial consortia could be the reason for their higher flexibility compared to more sensitive pure cultures [11]. However, it is still challenging to characterise complex biogas-producing microbial communities and elucidate the actual metabolic traits. Furthermore, problems regarding the handling and storage of microbial communities for scale-up often prevent their use in the practice [11].

Considering the fast progression in omics techniques, options for in-depth characterisation of complex microbial communities have evolved. In a recent study performed by Maus et al. *Petrimonas mucosa* ING2-E5A was isolated on a modified Columbia Agar Base medium from a mesophilic biogas reactor. Integrated omics analyses showed the enrichment of this species during unstable reactor conditions [12]. The study demonstrated the * Petrimonas* species’ role in anaerobic digestion processes, most likely regarding the fermentation of sugars and amino acids [12]. Although several more recent studies paid attention to the composition of microbial communities of BM processes, the composition of these microbial communities as well as the effect of disturbances on process performance should be analysed in more detail [9, 11]. Applying 16S rRNA gene amplicon sequencing, Logroño and colleagues analysed the functional resilience of biogas-producing microbial communities after H_2_ starvation in a batch process [13]. Wahid et al. also applied this technique to illustrate variations in the composition of microbial communities during discontinuous H_2_-feeding for an in situ biological upgrading of biogas reactors [9]. Both studies highlighted the significant role of hydrogenotrophic *Methanobacterium* species in an efficient BM process [9, 13]. Insights into microbiomes were further improved by applying metaproteomics to analyse both, the taxonomic and the functional composition of complex microbial communities [14]. Here, metaproteome data could be linked to certain reactor conditions, providing valuable insights for process optimisation [14]. Furthermore, combined metagenome and metaproteome approaches enabled to obtain a comprehensive view on the genetic potential and the metabolic activity of microbial communities [8].

The present study investigated BM process performance and robustness during H_2_ oscillations and applied discontinuous H_2_-feeding regimes to a laboratory-scale BM reactor running for more than 1.5 years during an experimental period of 70 days. The continuous-stirred tank reactor (CSTR) was operated at mesophilic (40 °C) condition and process variables such as pH value, gas composition, CO_2_ and H_2_ to CH_4_ conversion yields and CH_4_ production rate were thoroughly monitored. To characterise changes in the composition of the microbial community, integrated omics analyses were applied, which shed light on species interactions and adaptive mechanisms most likely linked to their resilience over inherent fluctuations during demand-oriented H_2_-feeding.

## Results

### Effects of discontinuous H_2_-feeding on process performance

**Fig. 1:**
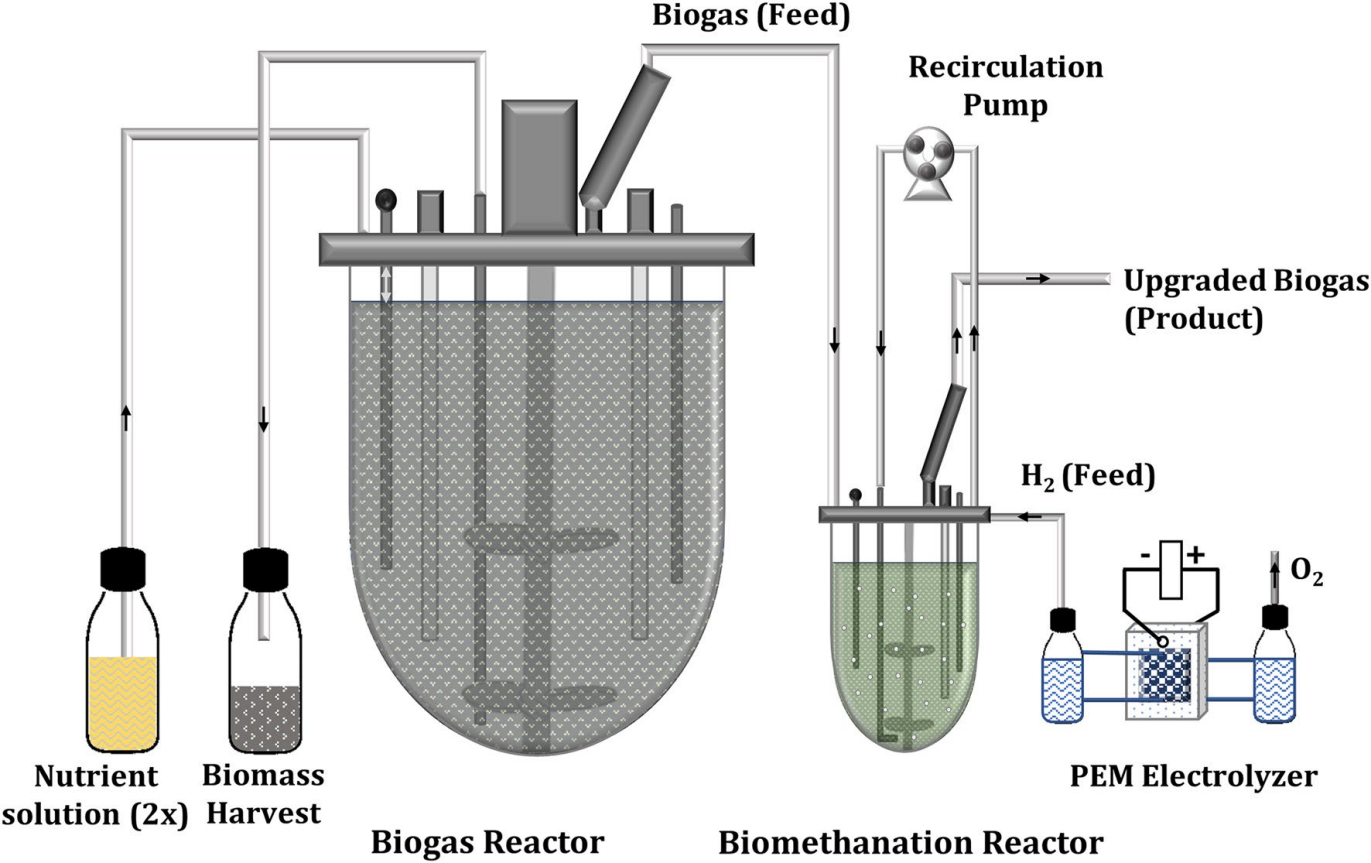
Schematics of the laboratory scale setup for the biological methanation of biogas. Hydrogen produced using a polymer electrolyte membrane (PEM) electrolyser

**Fig. 2:**
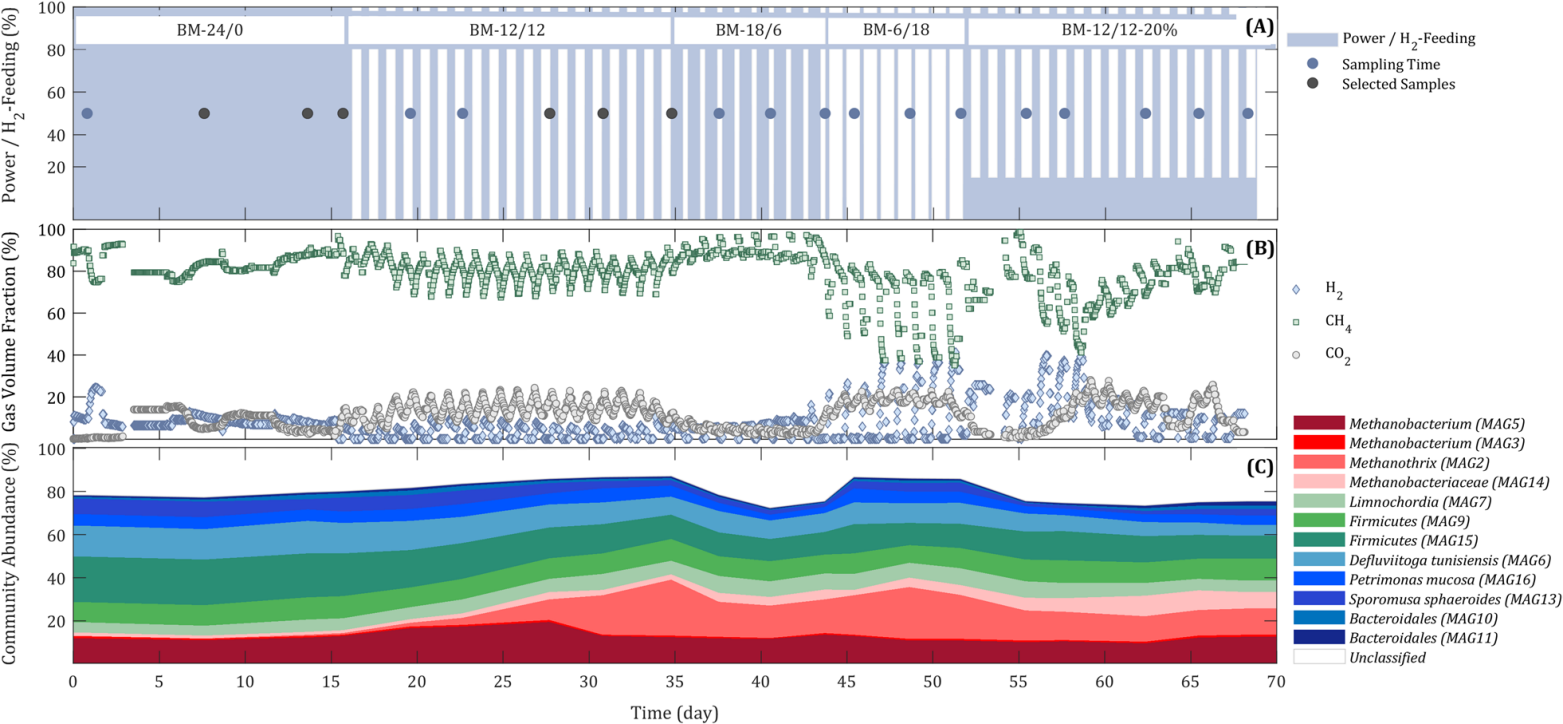
Time course of the biological methanation process during H_2_-feeding experiments. **A** H_2_-feeding regimes and sampling time of the biological methanation reactor; the selected samples were further used for statistical analysis, **B** volume fraction of the product gases, **C** community abundance. The unclassified taxon in the community composition, refers to the sum of spectral counts assigned to the low abundant metagenome-assembled genomes (MAGs) including uncategorised MAGs (MAG1 and MAG4) and MAG12 (*Lutispora*) and MAG8 (*Methanobacteriaceae*) and also the non-binned metagenome. See details of the microbial community in Additional file 1

To follow the BM process performance alterations with H_2_ oscillations, after one and half years of continuous biomethanation of CO_2_, which was provided by the biogas reactor (Fig. 1), demand-oriented H_2_-feeding experiments were started (Table 1). The intention was to mimic daily fluctuations that can occur in the electrical grids and to monitor the long-term effects of process alterations at the microbial level. Over the first 2 weeks (BM-24/0), H_2_ was fed continuously at 2.6 ${\mathrm{L}}_{{\mathrm{H}}_{2}}/{\mathrm{L}}_{\mathrm{MR}}/\mathrm{d}$ (H_2_ feeding rate: volume of H_2_ per volume of biomethanation reactor (MR) and day) and four biomass samples were collected for metaproteomics analysis. On average, the continuous process BM-24/0 (Fig. 2A, B) resulted in a daily maximum CH_4_ volume fraction in outlet gas (${\mathrm{y}}_{{\mathrm{CH}}_{4},\mathrm{ out},\mathrm{ max}}$) of 84.78 ± 3.34% and a CH_4_ production rate (MPR) of 0.71 ± 0.07 $({\mathrm{L}}_{{\mathrm{CH}}_{4}}/{\mathrm{L}}_{\mathrm{MR}}/\mathrm{d})$ (Table 1). The daily average of the relative conversion yield of H_2_ to CH_4_ (${\mathrm{Yrel}}_{{\mathrm{CH}}_{4}:{\mathrm{H}}_{2}}$) was 0.80 ± 0.08 and the daily average of absolute conversion yield of CO_2_ to CH_4_ (${\mathrm{Yabs}}_{{\mathrm{CH}}_{4} :{\mathrm{CO}}_{2}}$) was 73 ± 0.07 (Table 1).

**Table 1:** H_2_-feeding experiments and process parameters: maximum and minimum pH value, CH_4_ production rate (MPR), daily maximum CH_4_ volume fraction in the outlet gas${(y}_{{CH}_{4},out, max})$, daily relative conversion yield of H_2_ to CH_4_ (${Yrel}_{{CH}_{4}:{H}_{2}}$) and daily absolute conversion yield of CO_2_ to CH_4_ (${Yabs}_{{CH}_{4} :{CO}_{2}}$). Data with errors represent the standard deviation around the mean within each H_2_-feeding regime

H_2_-feeding regime (BM-On/Off)	pH _min_	pH _max_	$MPR$ $({L}_{{CH}_{4}}/{L}_{MR}/d)$	${y}_{{CH}_{4} ,out, max}$(%)	${Yrel}_{{CH}_{4}: {H}_{2}}$(${L}_{{CH}_{4}}/{L}_{{H}_{2}}$)	${Yabs}_{{CH}_{4} :{CO}_{2}}{(L}_{{CH}_{4}}/{L}_{{CO}_{2}}$)
BM-24/0	8.3	8.5	0.71 ± 0.07	84.78 ± 3.34	0.80 ± 0.08	0.73 ± 0.07
BM-12/12	7.9	8.9	0.59 ± 0.07	79.89 ± 1.76	0.67 ± 0.08	0.61 ± 0.07
BM-18/6	9.2	9.7	0.80 ± 0.07	88.33 ± 2.71	0.91 ± 0.08	0.82 ± 0.07
BM-6/18	8.8	9.2	0.43 ± 0.05	70.74 ± 3.94	0.49 ± 0.05	0.44 ± 0.05
BM-12/12–20%^a^	8.8	10.2^b^	0.66 ± 0.10	68.95 ± 8.01	0.75 ± 0.11	0.67 ± 0.10

^a^Taking into account lowering the nominal H_2_-feeding rate to 20% instead of a complete shutdown

^b^The pH value was measured on the day after an unintended interruption in CO_2_-feeding

Starting with discontinuous H_2_-feeding (Fig. 2A, B), the concentrations of CH_4_ fluctuated with oscillations of the H_2_ load. To evaluate the impact of these alterations on the microbial community, biomass samples were collected as indicated in Fig. 2A. Over the first days of BM-12/12, fluctuations appeared irregular. However, with progressing time, patterns became similar. Another visible disturbance in biogas production occurred during the switch between BM-6/18 to BM-12/12–20% (Fig. 2B). Here, the unexpected changes in the height of amplitudes were caused by a technical failure in feeding biogas to the BM reactor.

Process performance parameters, including MPR and conversion yields, decreased after switching to demand-oriented feeding from BM-24/0 to BM-12/12. The highest values of all parameters were measured for BM-18/6, which even exceeded those obtained for the continuous BM (BM-24/0). This could result from adaptations of the microbial community to the discontinuous H_2_-feeding regime favouring the enrichment of methanogenic *Archaea*, as will be shown later by metaproteomics of the microbial community.

A further increase in the off-time for the H_2_-feeding to 18 h (BM-6/18) resulted in the lowest performance compared to all other feeding regimes (Table 1). Starting with BM-12/12–20%, the process performance improved again. Except for CH_4_ purity, all other parameters were slightly increased compared to BM-12/12. Most likely, a basal feeding of H_2_ (20% of its nominal value) instead of a complete switch-off for 12 h provided minimum energy requirements of methanogenic *Archaea* in the microbial community, prevented more severe H_2_ starvation and delivered sufficient energy for maintenance metabolism.

During experiments, pH values were not controlled but only monitored. Starting with discontinuous feeding of H_2_, pH values oscillated with high values (maximum pH 10.2) during H_2_-feeding and lower values in non-H_2_-feeding phases (minimum pH 7.9). The oscillation was caused by capturing of CO_2_ from the liquid in H_2_-feeding phases and the accumulation of CO_2_ in the liquid during the non-H_2_-feeding phases. Potentially, these strong daily variations in pH values could challenge the stability of the microbial community.

As a result of discontinuous H_2_-feeding, the colour of the medium in the reactor changed from dark green–brown in feeding phases to light green–brown in non-feeding phases. The intensity of these oscillations was related to the duration of non-feeding phases and reached stability for longer than 12 h non-feeding phases. The dark colour of the liquid disappeared after a few hours when the biomass samples were exposed to air. Taken together, this can hint at the presence of a conductive media component, such as Fe minerals [15], in the electron transport chain.

### Microbial community structure as deduced from metagenome analysis

To gain insights into the taxonomic composition of the biogas-producing microbial community from the BM reactor, microbial metagenome sequencing followed by an assembly of sequence data and genome binning were applied. Obtained results were not only used to characterise the metabolic potential of the studied microbial community, but also to construct a database for subsequent metaproteome analysis. According to the literature, the generation of corresponding metagenome data for protein identification significantly increases the number of identified spectra [16].

A total of 2.8 Gbp metagenomic data were assembled and contigs were sorted into 16 metagenome-assembled genomes (MAGs); however, only 12 MAGs featuring metagenome completeness rates range from 80.0% to 98.3% and less than 5% contamination (Additional file 2: Tables S2.1 and S2.2). The Four MAGs with low completeness (MAG1, MAG4, MAG8, MAG12) were further not considered in this analysis and combined with the unclassified category, which explained below. A less diverse community structure was expected due to the fed substrate (H_2_ only). In total, 8 MAGs were assigned to the super kingdom *Bacteria* and 4 MAGs to the domain *Archaea*. At the phylum level, MAGs were allocated to the *Firmicutes* (MAG7, MAG9, MAG13, MAG15), *Bacteroidetes* (MAG10, MAG11, MAG16), *Thermotogae* (MAG6) and *Euryarchaeota* (MAG2, MAG3, MAG5, MAG14). Only few MAGs were classifiable at the family level (*Sporomusaceae* (MAG13), *Dysgonomonadaceae* (MAG16) *Petrotogaceae* (MAG6), *Methanotrichaceae* (MAG2), *Methanobacteriaceae* (MAG3, MAG5, MAG14), followed by the remaining five MAGs of the domain *Bacteria*, which were not classifiable to any other known family. Among the MAGs that were received, a total of five MAGs could be assigned to certain genera, namely, *Methanothrix* (MAG2), *Methanobacterium* (MAG3), *Defluviitoga* (MAG6), *Sporomusa* (MAG13) and *Petrimonas* (MAG16)). The remaining metagenome sequences, which were not binned and classifiable to any known taxon, were assigned to an unclassified category. The MAGs categorised in the same level of commonly known taxon were further differentiated in this text using MAG’s numbers in brackets, i.e., *Methanobacterium* (MAG5) and *Methanobacterium* (MAG3).

In the BM reactor, the bacterial MAG15 (9.7%, unclassified *Firmicutes*) was the predominant bacterial taxon, followed by MAG16 (2.8%) representing a member assigned to *Petrimons mucosa* species. Among methanogens, the archaeal MAG14 (20% of all metagenome sequences), MAG5 (18.5%) and MAG2 (7.5%) were abundant archaeal genomes with *Methanobacterium* (MAG14) and *Methanothrix* (MAG2) as the dominant genera. However, it must be considered that only metagenome data that could result in a MAG was assessed for further taxonomic, genome and protein analysis. In contrast, the remaining data most probably included other organisms which genomes we could not assemble.

### Microbial community structure as deduced from metaproteome analysis

#### Protein profiles

**Fig. 3:**
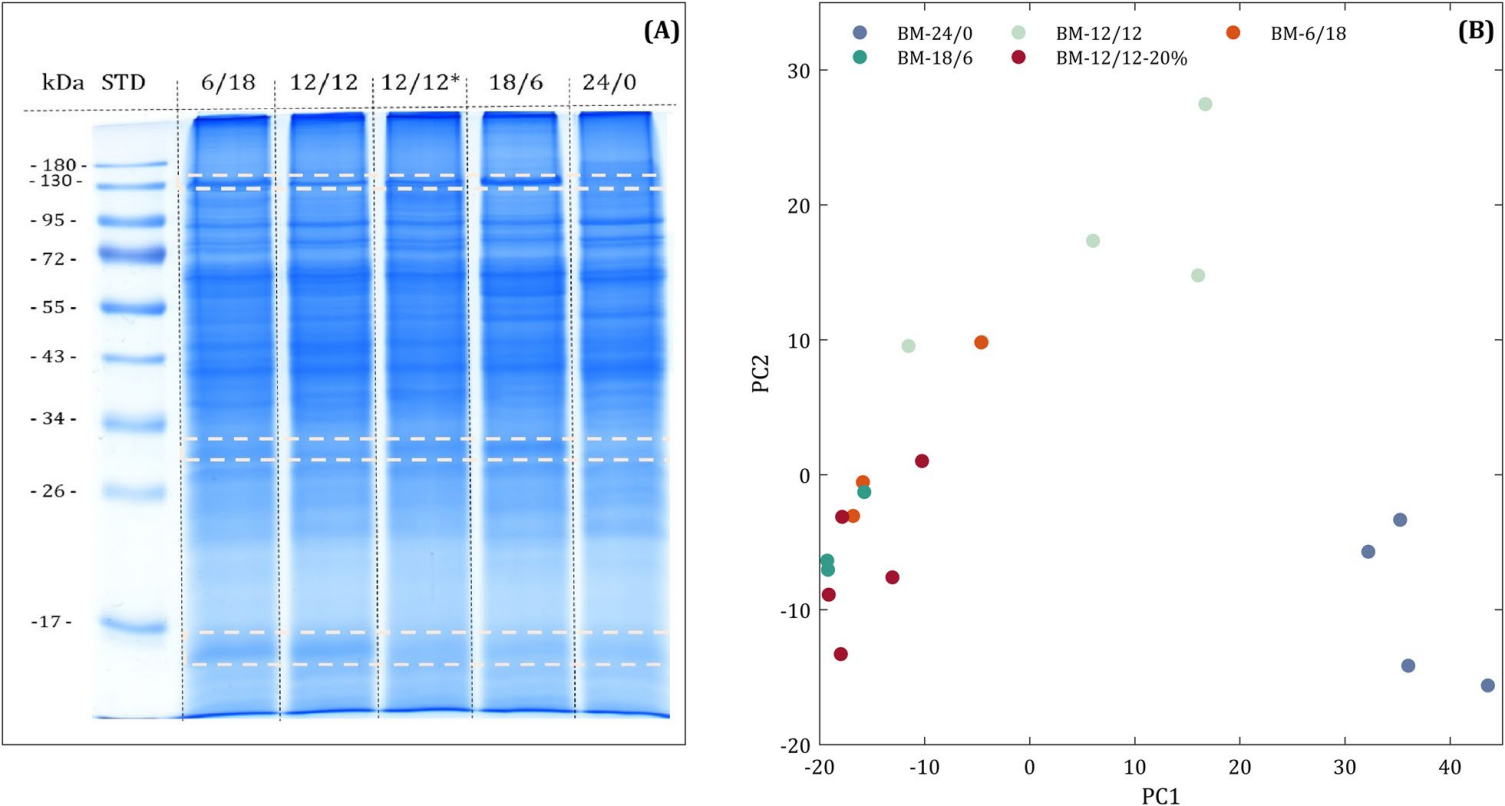
Protein profiles of the biological methanation reactor during discontinuous H_2_-feeding (**A**) Protein extracts (25 µg) of the third sample were loaded for sodium dodecyl sulphate–polyacrylamide gel electrophoresis. 12/12* shows the experiment with 20% of nominal H_2_-feeding (BM-12/12–20%). The white dashed lines enclose areas of visible variations in protein profiles. **B** Principal component analysis (PCA) plot of the 1000 most abundant metaproteins based on the Euclidean similarity index. The PCA plot reflects the development of microbial communities over the experiment period. See details of PCA plot generation in Additional file 4

Sodium dodecyl sulphate–polyacrylamide gel electrophoresis (SDS–PAGE) was carried out for the separation of all samples to obtain an overview of protein composition for the different H_2_-feeding regimes and for fractionation of samples before high-performance liquid chromatography–tandem mass spectrometry (HPLC–MS/MS) of tryptic peptides. Mass spectrometry (MS) results were used to characterise the microbial communities and identify alterations during discontinuous H_2_-feeding. The protein profiles of the SDS–PAGE (Fig. 3A) revealed the altered expression of proteins during discontinuous H_2_-feeding, whereas samples of one H_2_-feeding condition showed a nearly identical pattern (Additional file 3 Fig. S3.1).

To evaluate the effect of discontinuous feeding on the metaproteome, a principal component analysis (PCA) plot was compiled using the 1000 most abundant metaproteins (protein groups) of all samples. The samples represented each condition of the discontinuous H_2_-feeding (Fig. 2A), taken on different days. Although samples did not represent true biological replicates, they clustered more or less densely, indicating a high reproducibility of the metaproteome analysis. As expected, an obvious shift between BM-24/0 and experiments with discontinuous H_2_-feeding was identified.

Whereas samples of BM-18/6, BM-6/18 and BM-12/12–20% clustered closely, BM-12/12 was allocated between BM-24/0 and samples of the other discontinuous feeding regimes. This suggests that the adaption process of the community was still in progress for BM-12/12. This was also supported by the appearance and fading of the gel bands between samples of BM-12/12 (Additional file 3 Fig. S3.1B), whereas samples of other regimes were reproducible (Additional file 3 Fig. S3. 1A,C,D).

#### Community structure

The community composition during the H_2_-feeding experiments was additionally characterised using the abundance profile of proteins identified for each respective MAG. Approximately 80% of the all detected and taxonomically allocated detected metaproteins could be assigned to 12 MAGs (Fig. 2C, Tables S2.2 and S2.3). During discontinuous feeding of H_2_, the community shifted toward a higher abundance of *Archaea* (14% in BM-24/0, 27% in BM-12/12–20%), whereas the abundance of *Bacteria* decreased inversely. Taxonomical assignments of proteins at the order level displayed a strong increase in *Methansarcinales* (represented by the genus *Methanothrix*, Fig. 2C) and a slight increase in *Methanobacteriales*. In all discontinuous experiments with low H_2_-feeding time (BM-12/12 and BM-6/18), *Methanothrix* was increased compared to the experiments with high H_2_-feeding times (BM-18/6 and BM-12/12–20%). Here, a decrease in the bacterial community was noticeable for *Limnochordia*, *Defluviitoga tunisiensis and Sporomusa sphaeroides*.

#### Community functions

**Fig. 4:**
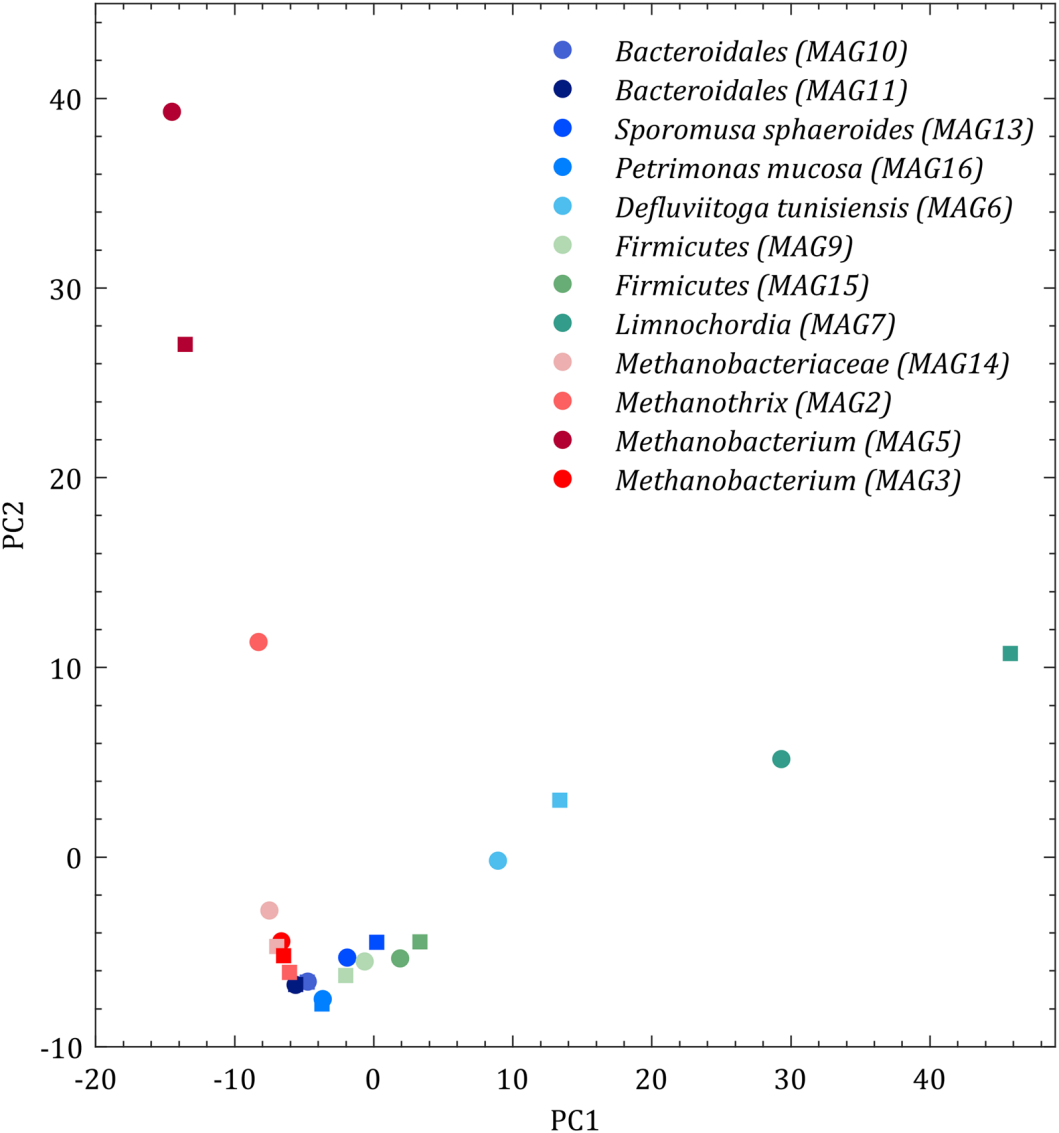
Principal component analysis (PCA) of the biogas microbiome to identify changes in the metabolic activity for two H_2_-feeding experiments (BM-24/0, filled rectangle and BM-12/12, filled circle). To avoid an overcrowded PCA plot, only the mean values of the samples of these two H_2_-feeding regimes are shown. See details of PCA plot data generation in Additional file 6

Functional analysis focusing on biochemical pathways related to the carbon and energy metabolism demonstrated the presence of glycolysis/gluconeogenesis and the reductive hexulose phosphate (RHP) pathway including carbon fixation with ribulose-1,5-bisphosphate-carboxylase/-oxygenase (RuBisCO) (Additional file 5, Fig. S5.1–13). Furthermore, the citric acid cycle, the Wood–Ljungdahl pathway and enzymes related to acetoclastic and hydrogenotrophic methanogenesis. For detailed metabolic analysis and characterisation of the main metabolic alterations, the metaproteins representing the functions were assigned to abundant MAGs of the continuous feeding regime BM-24/0 and the discontinuous feeding regime BM-12/12 (for details of pathways related to the MAGs, see the Kyoto Encyclopaedia of Genes and Genomes; KEGG maps in Additional file 5 Fig. S5.1–13). Based on the metabolic functions, MAGs could be separated by PCA into archaeal and bacterial sub-communities that are represented by the two clusters in Fig. 4.

**Fig. 5:**
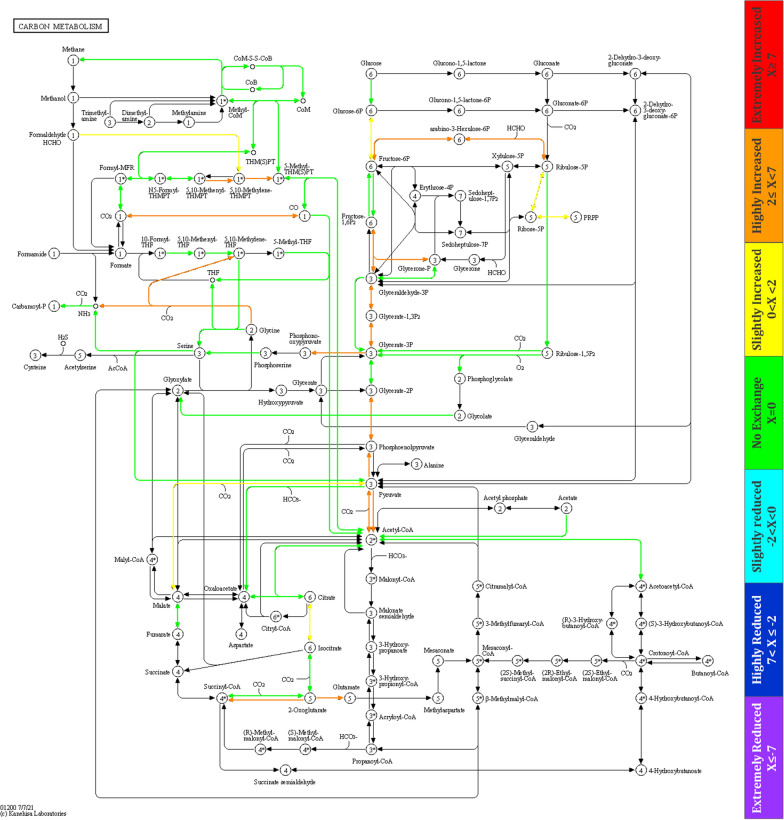
Differential expression of proteins in *Methanothrix* for BM-24/0 and BM-12/12 displayed in the Kyoto Encyclopaedia of Genes and Genomes (KEGG) map of the central carbon metabolism (map01200). Colours correspond to logarithmic expression ratio [X = Log_2_ (KO _BM-12/12_/KO _BM-24/0_)]. p > 0.05 considered significant, p ≤ 0.05 see colour legend at the right (*t* test). KO (KEGG Orthology) values are the mean normalised values of the abundance of defined orthologs in the metaproteins of the BM pattern. Further details of KEGG map data generation are available in Additional file 8

Larger distances between two samples of each MAG represent the difference of assigned metaproteins in continuous and discontinuous feeding and indicate that discontinuous feeding induced some metabolic adaptations for *Methanobacterium* (MAG5), *Methanothrix, D. tunisiensis and Limnochordia.* Besides its enrichment during discontinuous feeding, *Methanothrix* switched from acetoclastic to hydrogenotrophic methanogenesis (Fig. 5). Although the membrane-bound energy-conversion hydrogenase enzyme (Ech) is missing in this MAG’s associated metaproteins, other hydrogenase enzymes, e.g., F_420_-dependent hydrogenase (Frh) and membrane-bound viologen-reducing hydrogenase (Vht) and their fluctuation between experiments suggested an H_2_-uptake of *Methanothrix* (Additional file 7: Table S7.1). The presence of RuBisCo (Fig. 5) is most likely related to CO_2_ reduction in the RHP pathway, which has been recently shown to be involved in CO_2_ fixation in *Archaea* [17, 18]. RHP is further linked with methanogenesis in most of the methanogens using formaldehyde as an intermediate [17, 18] and was confirmed by the presence of the formaldehyde activating enzyme in the proteome of *Methanothrix*. The activity of *Methanobacterium* (MAG5), *Methanobacterium* (MAG2) and *Methanobacteriaceae* (MAG14) in hydrogenotrophic methanogenesis, was confirmed by detailed functional analysis (Additional file 5 Fig. S5.1–3). In addition, discontinuous H_2_-feeding seemed to have no or only minor effects on the metabolism of the other three methanogens. The fact that RuBisCO was not detected for these hydrogenotrophic methanogens underlines the specifics in the metabolism of *Methanothrix*. Results obtained for *S. sphaeroides* confirmed homo-acetogenesis activity and indicated that *S. sphaeroides* can compete with methanogen in the uptake of H_2_ and CO_2_ during H_2_-feeding. The capacity to synthesise or metabolise hydroxybutanoates could be related to the synthesis of polyhydroxyalkanoates as a storage compound that has been shown for *Veillonellaceae* isolated from compost samples [19, 20]. Most of the other identified proteins were assigned to pathways of the central metabolism. Expressed proteins were classified as belonging to the taxa of *Firmicutes*, *Bacteroidetes* and *Thermotogota*. Their central carbon/energy metabolism, showed their involvement in acidogenesis and acetogenesis via glycolysis/gluconeogenesis, pentose phosphate pathway, citric acid cycle, glycine/serine metabolism and fermentation pathways to acetate, propionate and butyrate (See Additional file 5 Fig. S5.5–13). Overall, the discontinuous H_2_-feeding regime seemed to have only a minor impact on the central carbon/energy metabolism of *Firmicutes*, *Bacteroidetes* and *Thermotogota.*

**Fig. 6:**
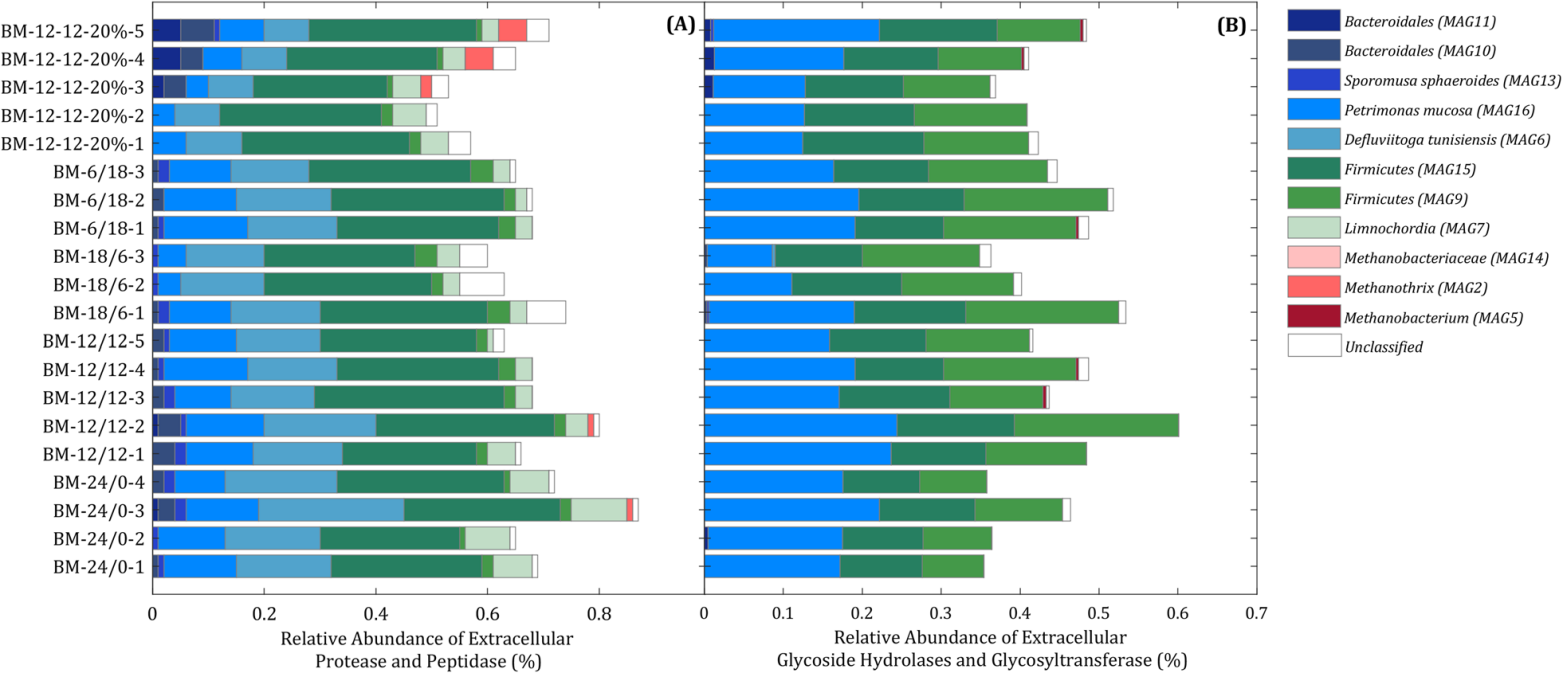
Relative abundance of extracellular proteolytic and hydrolytic enzymes for different samples of H_2_-feeding experiments. Extracellular secretion of (**A**) proteases and peptidases, (**B**) glycoside hydrolases and glycosyltransferases during H_2_-feeding experiments. The spectral counts were normalised based on the total spectral count for each sample. Further details for generation of the plots are available in Additional files 9 and 10

As already has been shown by metagenomic data in the literature, these bacterial species express genes coding for extracellular glycosyl hydrolase (GH) and glycosyltransferase (GT), protease and peptidase whose products were predicted to facilitate growth of these microorganisms on different sugar molecules and complex carbohydrates and proteins [8, 21]. Accordingly, for all enzymes classes mentioned above, abundant hits were obtained by metaproteomics. To distinguish extracellular from intracellular enzymes, the SignalP 6.0 server was applied [22]. While more diversity could be seen in the secretion of extracellular proteases and peptidases (Fig. 6A), the secretion of extracellular GH and GT was mostly restricted to *Firmicutes* and *P. mucosa* (Fig. 6B). The obtained results also indicated numerous extracellular proteases and peptidases expressed by the MAGs *Firmicutes* (MAG15), *P. mucosa* and *D. tunisiensis* (Fig. 6A). Furthermore, *D. tunisiensis* showed a high abundance of bacteriocin protein family assigned to Linocin_M18 (see Additional file 11). Besides lytic enzymes, antimicrobials and lysogenic bacteriophages could play an active role in the biomass turnover cycle [23]. Metaproteins, which are annotated even to the bacteriophages or involved in the antiviral archaeal or bacterial immune system (i.e., clustered regularly interspaced short palindromic repeats (CRISPR)) were also identified (see Additional file 11).

## Discussion

### Process resilience against discontinuous H_2_-feeding in biological methanation process

MPR and yields of the BM process depend on reactor configuration and process parameters. They are influenced by mass transport of H_2_ to the liquid medium as the rate-limiting step. The MPR obtained for the CSTR in this study is relatively low compared to other reactor types [24]. While further optimisation is feasible, this does not interfere with a more comprehensive characterisation of the adaption of a microbial community to a demand-orientated BM process. Overall, the fast recovery of MPR with discontinuous H_2_-feeding suggests robustness of the BM community. In particular, the rapid on-set of methanogenesis after non-H_2_-feeding phases showed the resilience of the microbial community. This process stage was characterised by a higher abundance of methanogenic *Archaea*. Monitoring of the MPR was also reported by Strübing et al. [10] for an anaerobic thermophilic trickle bed reactor with short (30 min) feeding breaks. However, longer interruptions (24 h) caused longer delays (60 min) in CH_4_ production of the reported system [10]. Obviously, short interruptions without damaging the BM community resulted in no delay in BM process response as described by Strübing et al. [10], whereas longer delays (1 and 4 weeks) required at least 1 week for full recovery of BM performance as mentioned by Braga Nan et al. [25]. Interruptions of several hours in our experiment showed no or only a minor delay of BM. Considering the typical daily based on/off patterns of the electrical grid, the process can be operated in a stable manner. Furthermore, potential damage due to longer interruptions of the power supply should be preventable by low-level feeding of the microbial community with H_2_.

Besides MPR, discontinuous H_2_-feeding influenced also other process parameters as well as the composition of the microbial community. Variations in the pH value are caused by the accumulation of CO_2_ (acidification) in the medium during H_2_-non-feeding phases or by consumption of CO_2_ (alkalisation) during H_2_-feeding phases, since CO_2_ is delivered continuously with the biogas feed to the BM reactor. However, the production of volatile organic acids produced by acidogenesis/acetogenesis cannot be ruled out as a reason for a decrease in pH value, since fermentation pathways of acetate, propionate and butyrate were confirmed by the metaproteomic analysis. Decreasing pH values have been also related to concomitant homo-acetogenesis by Logroño et al. [26] in another study. In our setup, an increase in pH value could be a sign that hydrogenotrophic methanogenesis was out-competing homo-acetogenesis. The trend toward higher pH values (pH > 9.0) (Table 1) has been shown to favour hydrogenotrophic versus acetoclastic methanogenesis [27, 28], since acetoclastic methanogenesis depends on an acetate transporter, which consumes surplus energy for acetate uptake [27].

### The metabolism of *Methanothrix* in the enriched biological methanation culture

The increase of *Methanothrix* species was the major change within the microbial community during discontinuous H_2_-feeding. Previously described as a strictly acetoclastic methanogen [29], its ability for direct interspecies electron transfer (DIET) to CO_2_ has been shown in defined co-cultures with *Geobacter metallireducens*, recently [30, 31]. All genes related to the CO_2_ pathway involved in reduction to CH_4_ were also identified in the metagenome as has been shown for the reconstructed central carbon metabolism pathway using KEGG pathway tools (Additional file 12 Fig. S12.1). Interestingly, the identified proteins covering all enzymes of hydrogenotrophic and acetoclastic pathways are in good agreement with metatranscriptomic data of *Methanothrix* originating from rice paddy soil enrichment cultures [17], accepting electrons from *Geobacter* species producing e-pilins for DIET as the electron donor for hydrogenotrophic methanogenesis. Neither *Geobacter* species producing e-pilins nor multi-haems soluble cytochrome c were detected in the BM community, raising questions about the mode of electron transfer [31, 32]. The detection of the Frh and Vht indicated *Methanothrix* could probably accept H_2_ as an electron donor for methanogenesis. The presence of the complete RHP pathway has been demonstrated recently to act in CO_2_ fixation in *Archaea*. It was further linked to *Methanothrix concilii* with methanogenesis via formaldehyde as an intermediate, allowing an additional reduction of CO_2_ and possibly increased energy gain [17, 18].

More recent data for acetoclastic methanogenesis showed that *Methanosarcina mazei* can reduce Fe^3+^ as an electron acceptor during acetoclastic methanogenesis using the hydrogenotrophic pathway in reverse direction [33] and thereby increasing the ATP gain [34]. It is possible that a similar pathway in *Methanothrix* explains the presence of enzymes involved in hydrogenotrophic methanogenesis. The reduction of Fe^3+^ could cause the production of reduced iron minerals such as magnetite that could accumulate on the surface of methanogens [15]. Accordingly, the dark colour of the biomass during feeding periods could be due to the formation of magnetite. In contrast, the discolouration during non-feeding periods (data did not showed) could be related to oxidation of Fe^2+^ from magnetite delivering additional electrons for methanogenesis [15]. Furthermore, magnetite itself is a conductive mineral and has been shown to compensate for the deficiency of e-pilins, enabling probably direct electron transfer from fermenting organisms to methanogens [32, 35, 36].

Altogether, *Methanothrix* is an acetoclastic methanogen that probably uses H_2_ or external electrons as an additional electron source for methanogenesis. Also, it is reasonable that iron minerals could function as a sink or source of electrons depending on the respective thermodynamic conditions of the environment [15].

### The microbial food web in the enriched biological methanation culture

**Fig. 7:**
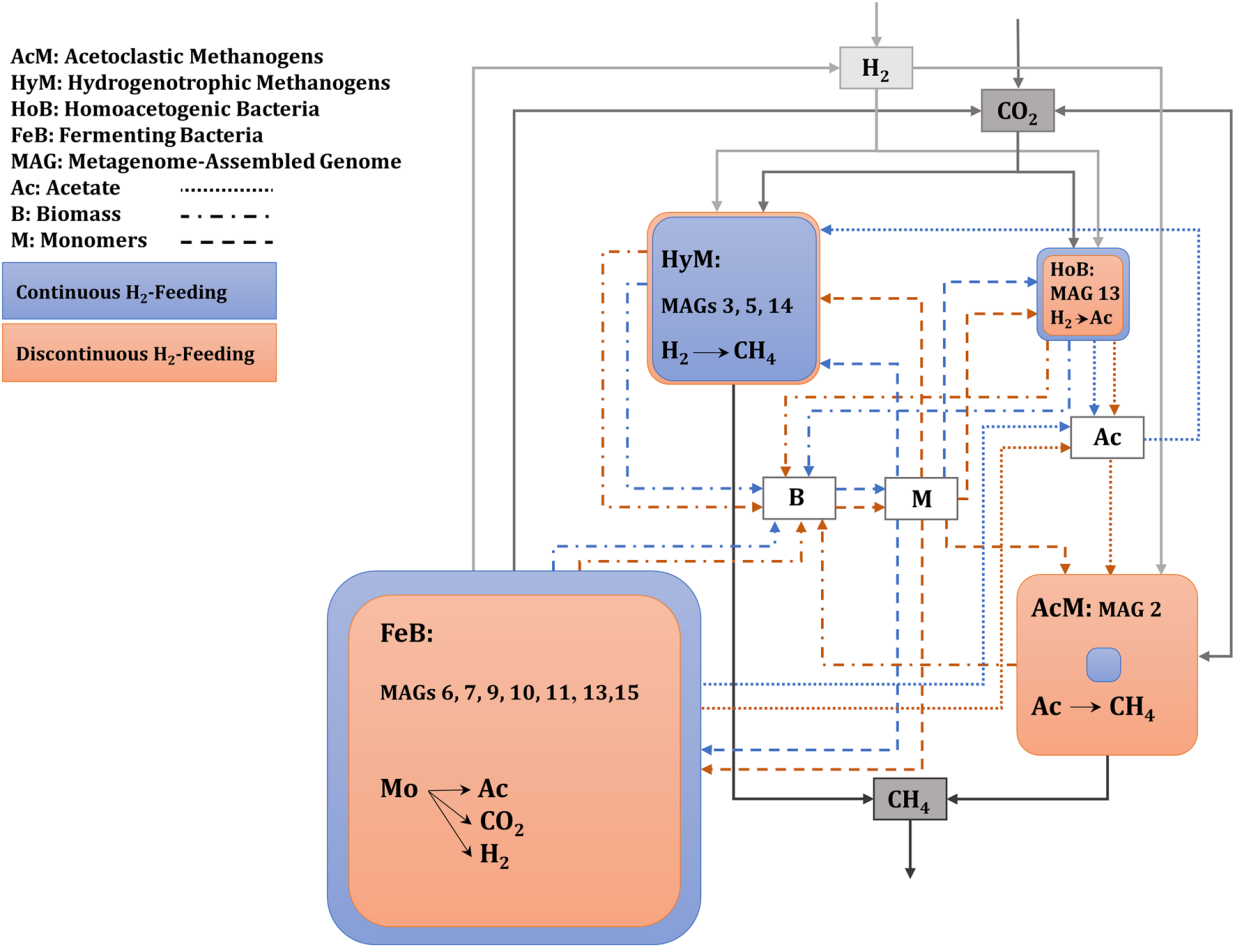
Microbial food web of a biological methanation (BM) process considering continuous H_2_-feeding (BM-24/0, blue) and discontinuous H_2_-feeding (BM-12/12, orange) experiments including in-/organic substrates and intermediates. The size of the boxes represents the abundance of the microbial groups of the last sample of the aforementioned feeding regimes. The full grey arrows depict the main substrate and product of the hydrogenotrophic BM process, whereas the other type of lines (BM-24/0, blue; BM-12/12, orange) are assigned to biomass (dash–dot), monomers/intermediates (dash) and acetate (dot)

Structural and functional analysis of proteome data revealed a microbial food web consisting of primary producers and consumers of biomass or small organic intermediates (Fig. 7). Hydrogenotrophic methanogenesis by *Methanobacteria* and homo-acetogenesis by *S. sphaeroides* feeding on H_2_ and CO_2_ were the pathways of primary production yielding not only the desired product (CH_4_) but also the side products biomass and acetate. The low proportion of biomass for *Methanobacteriaceae* as primary producer was surprising and did not correspond with the higher abundance of *Methanobacteriaceae* in the metagenome.

Continuing the food web, the high pH values of the medium indicated that the produced acetate is consumed continuously. During discontinuous H_2_-feeding, the abundance of the acetoclastic methanogen *Methanothrix* exceeded those for *Methanobacteria,* raising the total abundance of methanogens to about 40%. Whereas the prevalence of *Methanothrix* during discontinuous H_2_-feeding accounted for the acetate consumption, its low abundance during continuous H_2_-feeding could probably not explain overall acetate consumption to maintain the alkaline pH of the medium. In the absence of alternative consumers, such as syntrophic acetate oxidising *Bacteria*, the acetate could be assimilated by hydrogenotrophic methanogens as described before [37]. Moreover, some strains require the addition of acetate as a carbon source [37, 38] and the assimilation of acetate into biomass by hydrogenotrophic methanogens was also confirmed [39]. Higher acetate assimilation into biomass was detected by Oberlies et al. when the *Methanobacterium thermoautotrophicum* cells encountered a limited supply of H_2_ or CO_2_[39]. This could be beneficial due to spare in H_2_ and CO_2_[39]. Completing the food web, the participation of abundant *Bacteria* besides homo-acetogenic *S. sphaeroides* has to be elucidated. The only available carbon and energy source was the biomass produced by methanogenic *Archaea* and of homo-acetogenic *S. sphaeroides*. Due to strictly anaerobic conditions and missing alternative electron acceptors, primary and secondary fermentations remain as optional pathways for energy metabolism. *Bacteria* of the phyla *Firmicutes*, *Bacteroidetes and Thermotogota* identified by metagenome sequencing and metaproteomics matched those requirements. Furthermore, highly expressed extracellular GH, GT, peptidases and proteases seem to allow consumption of dead or living biomass of other community members, including the primary producers. While secretion of proteolytic enzymes was observed for *Firmicutes, P. mucosa, Defluviitoga tunisiensis, Limnochordia* and *Bacteriodales* in the present study, only *Firmicutes* and *P. mucosa* excreted GH and GT. Both, hydrolysis of pseudomurein of *Methanobacteriaceae* [40] and cellular proteins, sugars and amino acids released would allow bacterial growth, by fermenting these substrates to acetate, CO_2_ and H_2_, which are resubmitted to methanogenesis. The proposed microbial food web in this study is in agreement with closed nutrient recycling via microbial catabolism in the BM process of another study. By implementing real-time quantitative reverse transcription method, Savvas et al. showed the active role of bacterial substratum in redistribution of nutrients [41]. Besides lytic enzymes, detection of *Caudoviricetes* peptides, a species that specifically infects *Methanobacterium*, and the presence of other bacteriophage peptides suggest a decisive role in host–phage interactions in shaping the BM microbial community. However, more comprehensive studies are needed to clarify the role of phage–host interactions in cell lysis [23]. Linocin_M18, a bacteriocin peptide that was highly expressed by *Defluviitoga tunisiensis*, may be involved in proteolytic activities. It has also been reported to inhibit the growth of gram-positive bacteria [42]. Oxidative stress response in anaerobic environment could be another explanation for the high expression of linocin-like proteins [43]. A high abundance of *P. mucosa* harbouring GH enzymes and peptidases was correlated to disturbed full-scale agricultural biogas plants [12]. This species was predicted to cope with corresponding stress conditions and displaced other competing *Bacteria* that are less stress resistant [12]. Most likely, its abundance is related to feeding on microbial biomass. The presence of *Limnochordia*, which did not seem to express any hydrolytic enzymes, indicated that it was cheating at monomeric substrates uptake. Most likely, this characteristic is not restricted to bacterial community members, since many methanogens are dependent on external amino acids or their growth is at least stimulated in the presence of amino acids [37, 41]. Taken together, the metabolic food web discussed seems to provide a reasonable explanation for the stability of the co-culture of methanogens with heterotrophic *Bacteria*. However, the low abundance of methanogens during continuous feeding is still surprising. Considering the ATP gain in catabolism and the expenses for biosynthesis, methanogens as well as homo-acetogens with their lower energy gain and higher expenses for biosynthesis are at a disadvantage compared to many other fermenters. This means that a high turnover of substrates is required for the synthesis of a small amount of cell biomass, as has been shown for syntrophic acetate oxidizing *Bacteria* in anaerobic digestion [44]. For a community in steady state, this results in a lower abundance compared to others. Another reason for the low abundance of methanogens could be a high turnover of the biomass of the methanogens due to degrading enzymes released by *Bacteria* or bacteriophage-induced cell lysis providing sufficient nutrients for the increase in bacterial biomass. Consequently, higher growth rates of methanogens should compensate for cell lysis, which could be easily achieved at a dilution rate of 0.004 (1/d). Mathematical models that are under development should allow for the testing of the corresponding hypotheses [45].

During discontinuous feeding, the microbial community adapted to a higher abundance of methanogenic *Archaea*, which is in agreement with recent reports [9, 13, 46]. *Methanobacterium* was found as the most abundant member. Its presence also seemed crucial for stabilising the MPR of the system after H_2_ interruptions [9, 47]. Whereas the abundance of *Methanobacteria* remained nearly the same during discontinuous H_2_-feeding experiments, a drastic increase of *Methanothrix* after the on-set of discontinuous H_2_-feeding was observed. According to the reconstructed microbial food web, *Methanothrix* consumed acetate. On the one hand, the interruption of H_2_-feeding most likely stopped acetate production by *S. sphaeroides*. On the other hand, acetate, H_2_ and CO_2_ were still released from the degradation of microbial biomass in non-feeding periods. Under these conditions, assimilation of acetate by hydrogenotrophic methanogens is limited by a low H_2_ supply for methanogenesis causing energy constraints. Therefore, *Methanothrix* seemed to benefit from the remaining acetate supply. Furthermore, the acetate consumption by *Methanothrix* prevented acidification during non-feeding periods. The increased expression of enzymes involved in hydrogenotrophic methanogenesis suggests that *Methanothrix* is competing for H_2_ via hydrogenases or electrons via DIET during H_2_-feeding. In contrast, the abundance of most bacterial community members was slightly decreased during discontinuous feeding. This could be related to a reduced production of biomass as a substrate, whereas the stable abundance of *Firmicutes* might be related to their hydrolytic activity.

## Conclusions

Taken together, the stability of demand-oriented BM processes and the ability of self-regenerating microbial communities to adapt to discontinuous feeding regimes support power to methane scenarios. Some functions of the microbial food web, particularly the ability of microorganisms to compensate for process deficiencies and persist during power-related oscillations, could be considered advantageous for the operation of discontinuous BM processes. This is characterised by: (i) homo-acetogenic members that produce acetate, which is assimilated by hydrogenotrophic methanogens facilitating their growth. (ii) Hydrolytic *Bacteria* that recycle biomass and provide essential nutrients that are assimilated by methanogens. (iii) H_2_ released as a side product of bacterial fermentation, which delivers essential reducing power for the maintenance of hydrogenotrophic methanogens during non-feeding periods. (iv) Acetoclastic methanogens maintaining the pH value during non-feeding periods by consuming acetate released from biomass recycling that hydrogenotrophic methanogens could not assimilate. Considering technical applications, the nutrient cycling of microbial communities makes the addition of complex external substrates unnecessary. Furthermore, the metabolic heterogeneity compared to a pure culture seems to optimise metabolic pathways and attenuate the effects of discontinuous H_2_-feeding. Overall, the results underline the potential of BM to convert VRERs into biomethane for long-term storage and long-distance transport in the gas-grid. Moreover, immediate switching-off without damage to the microbiome as a biocatalyst could contribute to grid stability during dark doldrums.

## Materials and methods

### Chemicals and nutrient solutions

All chemicals used in this study were at least of analytical grade; for LC–MS/MS, MS-grade solvents were used. The composition of all nutrient solutions used for the growth of microorganisms is found in Table S13.1, Additional file 13.

### Inoculum preparation

As inoculum, the fresh biomass from a local biogas plant (Magdeburg, Germany) was used. A detailed description of the preparation of the inoculation and the operation of the bioreactor system is given in Additional file 13, S13-1. Briefly, to adapt the fresh biomass to laboratory conditions, a small reactor that was operated continuously was inoculated with 400 mL of the fresh biomass. To keep the pH value of the reactor higher than 7.0, the feeding rate was increased from 1 mL/d until it reached a maximum of 20 mL/d (1x-nutrient solution with 11.5 g/L glucose as a carbon source). Afterward, a scale-up was done with a maximum feeding rate of 48 mL/d (1x-nutrients solution) in other reactor with 1 L working volume, 40 °C, 50 rpm, pH > 6,9. Finally, the obtained biomass was used as a preculture for inoculating a biogas reactor (2.1 L working volume) operated at a stable feeding rate of 48 mL/d (2x-nutrient solution). For details, see “Biogas reactor” below.

### Experimental setup and operation conditions

As depicted schematically in Fig. 1, the setup mainly consisted of two CSTR and a water electrolyser. The primary CSTR (biogas reactor; see below) was operated to produce continuously biogas (source of CO_2_) as one of the main substrates for the second CSTR (BM reactor; see below), where the consortium of microorganisms enriched from the agricultural biogas plant converted CO_2_ and H_2_ to CH_4_. Hydrogen as the second main substrate of the BM was provided by direct electrolysis of water using a polymer electrolyte membrane (PEM) electrolyser. A pump was used for the second reactor to recirculate the headspace gas to increase the mass transfer of H_2_ and CO_2_ to the liquid medium. For details see “Biological methanation reactor” below.

#### Biogas reactor

The biogas reactor, a stainless-steel dish bottom vessel (volume 2.5 L) was controlled via a BioFlo^®^320 system (Eppendorf, Inc., Enfield, CT, USA). The active culture (2.1 L working volume) was stirred continuously (75 rpm) with using two marine blade impellers (outer diameter 6.5 cm) placed at the height of 67 and 105 mm. The temperature was controlled at 40 °C using a Pt100 probe. The pH value and oxidation–reduction potential (ORP) were monitored using pH and ORP ISM^®^ sensors (Inpro 325X (i), Mettler Toledo^®^, Geissen, Germany); operation conditions: 7 < pH < 8 and, -350 < ORP < -450. Nutrient feeding and biomass harvest were done by a pulse-feeding method using BioCommand^®^ (Eppendorf, Inc., Enfield, CT, USA) using a peristaltic pump (48 mL/d; 2x-nutrient solution containing 23 g/L glucose) to keep the volume of the reactor at a fixed level. All remaining reactor ports were closed either by stainless steel plugs or connected to gastight tubing closed with a luer/lock sampling valve (Eppendorf AG, Hamburg, Germany). The produced biogas was passed through a gas condenser to remove water vapour and transferred through gastight tubing (Santoprene® LEZ-SAN, internal diameter: 1.6 mm, thickness: 1.6 mm, Medorex, Nörten-Hardenberg, Germany) and a one-way valve port to the BM reactor for further processing of the CO_2_ of the biogas to biomethane.

#### Biological methanation reactor

The BM reactor in Fig. 1 (500 mL vessel, Sixfors multi bioreactor system, INFORS AG, Switzerland) was inoculated with biomass collected from the preculture reactor (Additional file 13, S13-1). The BM reactor, containing enriched hydrogenotrophic methanogens, was continuously running for more than 1.5 years before discontinuous H_2_-feeding. The cultivation conditions were controlled with an integrated Pt100 temperature probe and a pH sensor (Type 405-DPAS-SC-K8S, Mettler-Toledo GmbH, Giessen, Germany). The temperature was controlled at 40 °C; the pH value varied between 6.9 to 10.2 depending on the feeding condition. To adapt microorganisms to shear stress, agitation was increased stepwise (each step 100 rpm) until 800 rpm employing a magnetic propeller stirrer (INFORS AG, Bottmingen, Switzerland). H_2_ and biogas were added through a one-way valve to the headspace and the headspace gas recirculated using a peristaltic pump 200 rpm (Watson-Marlow 120U/DV, Falmouth, Cornwall, United Kingdom) using gastight tubing. An exhaust gas cooler supplied with 20 °C cold water (Minichiller 300, Huber, Berching, Germany) was used to remove aqueous vapour from the upgraded biogas. When the reactor pressure reached 80 mbar, the headspace was vented automatically and the exhaust gas analysed using a gas analysis system (GärOnA, Gesellschaft zur Förderung von Medizin-, Bio- und Umwelttechnologien e. V. (GMBU), Halle, Germany; mobilGC Elektrochemie Halle GmbH (ECH), Halle, Germany). All unused reactor ports were closed either by plugs or by gastight tubing closed with a luer/lock sampling valve. Nutrient solution (1x) was fed after each sampling per hand (12 mL/week).

#### Discontinuous H_2_-feeding and sampling

Starting from stable process conditions (see Fig. 2A), the BM reactor was exposed to a discontinuous H_2_-feeding regime for about 70 days. Therefore, the scheduled power supply provided different electricity loads for the water electrolyser. Each loading pattern was repeated for several days to collect samples for microbial community analysis. The gas composition was monitored online using the “GärOnA” gas analysis system. The collected biomass samples were centrifuged (10 min, 10,000 × g, room temperature) and the pellets stored at − 20 °C. The H_2_-feeding regimes and the sampling times are shown in Fig. 2A starting with the first sampling timepoint (0 d) at continuous H_2_-feeding (BM-24/0). With continuous feeding, the experiment proceeded with 12 h (BM-12/12), 18 h (BM-18/6) and 6 h (BM-6/18) H_2_-feeding regimes. Finally, it ended with 12 h H_2_-feeding regime and 20% of the nominal feeding during the off-phase (BM-12/12–20%).

### Gas chromatography

Gas composition and volumes were analysed in real time using a “GärOnA” system with an integrated gas chromatography (GC) analysis instrument. Therefore, the exhaust gas cooler of the BM setup was connected to the “GärOnA” system. The gas pressure was measured by a sensor head as part of the “GärOnA” system. At a pressure of 1080 mbar, the gas was released automatically via gastight tubing from the reactor headspace to a 50 µL sample loop (operated at 40 °C) of the GC system. High purity argon gas (Argon 5.0; Westfalen AG, Münster, Germany) at the pressure of 2.5 bar was used as the carrier through the stainless-steel GC column (ShinCarbon ST Micropacked GC column operated at 180 °C, Restek Corporation, Bellefonte, PA, USA). The amount of H_2_, CH_4_ and CO_2_ was determined with a thermal conductivity detector (TCD) coupled with the GC system [48]. The produced gas volume was calculated using the software of the “GärOnA” system, taking into account the pressure and the headspace volume of the BM system.

### Process parameter estimations

Assuming a stoichiometry of 1 CO_2_ + 4 H_2_ ⟶1 CH_4_ + 2 H_2_O for BM, the process performance was evaluated using the following equations. The volume fraction of the different gases *i* in the input and the output ${(y}_{i})$ were calculated as

1 $${y}_{i}=\frac{{V}_{i} {out}}{{V}_{T} {out}} \left(\frac{{L}_{i}}{{L}_{T}}\right)\times 100 \left(\%\right)$$

where ${V}_{i} {out}$ is the volume of component i (L) in the outlet gas and ${V}_{T} {out}$ (L) is the total of volume of outlet gas in the normal condition.

The MPR provides information on the productivity of the reactor, which is normalised based on the MR volume (V_MR_) as follows:

2 $$MPR =\frac{{V}_{CH_{4} {out}}-{V}_{CH_{4} {in}} }{\left({V}_{MR}\times t\right)} \left(\frac{L _{{CH}_{4}}}{ \, {L}_{MR}\times d}\right)$$

where ${V}_{{CH}_{4} out}$ (L) is equal to the total volume of CH_4_ in the outlet, ${V}_{{CH}_{4} {in}}$ (L) is the total volume of CH_4_ in the inlet, V_MR_ (L) is the working volume of the reactor and t (d) represents the time [49].

The absolute CO_2_ conversion yield CO_2_, ${Yabs}_{CH4:CO2}$ gives information about the efficiency of CO_2_ conversion to CH_4_ and was calculated as [49]

3 $${Yabs}_{CH_4:CO_2} =\frac{{V}_{CH_{4} {out}}-{V}_{CH_{4} {in}}}{{V}_{CO_{2} {in}} } \left(\frac{L _{{CH}_{4}}}{L _{{CO}_{2}}}\right)$$

where ${V}_{CO_{2} {in}}$ (L) is the volume of input CO_2_ in the process.

The absolute conversion yield of H_2_, ${(\mathrm{Yabs}}_{{CH}_{4}:{H}_{2}})$ presented in Eq. 4 provides information about how much power is needed to produce a certain amount of CH_4_. The relative conversion yield ${\mathrm{Y}rel}_{{CH}_{4}:{H}_{2}}$ (Eq. 5) gives information about the efficiency of H_2_ conversion to CH_4_ and is obtained by division of the ${Yabs}_{CH_{4}:H_2}$ by 0.25 for a range 0 to 100% [49]:

4 $${Yabs}_{CH_{4}:H_{2}}=\frac{{V}_{CH_{4} {out}}-{V}_{CH_{4}{in}}}{{V}_{H_{2} {in}}} \left(\frac{L _{{CH}_{4}}}{L _{{H}_{2}}}\right)$$

5 $${Yrel}_{{CH}_{4}:{H}_{2}}=\frac{{\Upsilon }_{{CH}_{4}:{H}_{2}}}{0.25} \left(\frac{L _{{CH}_{4}}}{L _{{H}_{2}}}\right)$$

where the ${V}_{{H}_{2}in}$ (L) is the volume of input H_2_ in the process. The H_2_ production by water PEM electrolyser calculated based on Faraday’s law and is presented in Additional File 15, eq. S15-1to eq. S15-4.

### Microbial community analysis

#### Metagenome sequencing

Extraction of microbial genomic DNA, microbial metagenome library preparation and sequencing

The total DNA of microbial communities was extracted from BM digestates applying the NucleoSpin Microbial DNA Mini kit (Macherey–Nagel GmbH & Co. KG, Düren, Germany) according to the manufacturer’s instructions. RN’ contaminating in DNA samples was removed by adding 1 µL of RNase A (10 mg/ml, ThermoFisher Scientific, Dreieich, Germany) and incubation for 1 h at room temperature. For preparation of the sequencing library, DNA samples were purified using the Genomic DNA Clean & Concentrator Kit (Zymo Research, Irvine, CA, USA).

For sequencing on the Illumina platform, 1 µg of total DNA was sheared to approximately 430 bp fragments using a Focused-Ultrasonicator (Covaris M220, Woburn, MA, USA). Finally, the Illumina TruSeq Shotgun PCR-free Library sample preparation kit (Illumina, Eindhoven, Netherlands) was used to construct the sequencing library, which was sequenced on the Illumina MiSeq sequencer using the Illumina MiSeq Reagent Kit v3 (Illumina, Eindhoven, Netherlands), following a 2 × 300 indexed high output run protocol.

Megahit (v1.2.9) [50] tool was used for assembly of the sequencing data applying a range of k-mer sizes of 21, 31, 41, 51, 61, 71, 81, 91, 99. Subsequently, all sequencing reads were aligned to the assembled contigs with BBMap (v38.86) [51]. To convert the sequence alignment/map format (SAM) to binary alignment/map (BAM), sort the alignment file and calculate read mapping statistics, SAMtools (v1.3.1) [52] was used. Furthermore, to predict genes on assembled contigs larger than 1 kb, the gene prediction tool Prodigal v.2.6.3 [53] was applied. Predicted protein sequences were compared to the National Center for Biotechnology Information NCBI-nr database using the BLASTP mode of DIAMOND (v0.9.36) [54]. The resulting output file was loaded into MEGAN [55] for a taxonomic classification of each protein sequence. In addition, a taxonomic classification for each MAG was derived using the Genome Taxonomy Database (GTDB) toolkit [56].

The MetaBAT tool (v2.12.1) [57] was used to cluster the metagenome assemblies into the MAGs. Subsequently, completeness, contamination and strain heterogeneity of the MAGs were estimated with CheckM (v1.0.124) [58], using sets of clade-specific single-copy marker genes.

#### Metaproteomics workflow

##### Extraction of proteins, protein quantification and separation

A metaproteomics workflow established by Heyer et al. [59] was applied to characterise the microbial community of the BM process during the H_2_-feeding experiments. The metaproteomic workflow followed the techniques described in [14, 59, 60]. Briefly, a phenol extraction method using a ball-mill was applied to disrupt cells and extract proteins [60]. Proteins were precipitated with ice-cold 100 mM ammonium acetate in methanol, resuspended in urea buffer (7 M urea, 2 M thiourea and 0.01 g/mL dithiothreitol) and quantified with an amido-black assay [60]. Extraction of proteins (25 µg) from urea buffer was done with ice-cold 100% acetone overnight at − 20 °C; proteins were separated by 12% SDS–PAGE method [60]. Gels were stained with colloidal Coomassie overnight. To extract proteins from the gels, lanes were divided into ten equal slices and processed by tryptic in-gel digestion presented as a new workflow in the “B6” step by Heyer et al. [60]. Before LC–MS/MS measurement, the vacuum-dried peptides were resuspended in 30 μL of chromatographic mobile phase A (LC–MS water, 0.1% trifluoroacetic acid). Of each sample, the solution was subsequently centrifuged for 30 min, 4 °C, at 13,000 × g and 28 μL transferred into an HPLC vial.

##### Protein identification by mass spectrometry

Five microliters of the sample peptides were injected and separated using a liquid chromatography (LC) system (UltiMate® 3000 nano splitless reversed-phase nano HPLC; Thermo Fisher Scientific, Dreieich) equipped with a reversed-phase trap column (nano trap cartridge, 300 μm inner diameter × 5 mm; packed with Acclaim PepMap100 C18, 5 μm particle size, 100 Å pore size, nanoViper, Bremen, Germany) and a reversed-phase separation column packed with (Acclaim PepMap RSLC C18, 2 μm particle size, 100 Å pore size, 75 μm inner diameter and 500 mm length, Bremen, Germany). The separation was started, where the gradient was 5 to 35% mobile phase B (acetonitrile, 0.1% formic acid, 99%) over 120 min at a flow rate of 0.4 mL/min. The LC was coupled online to a timsTOF™ Pro mass spectrometer (Bruker Daltonik GmbH, Bremen). The timsTOF™ Pro was equipped with a captive spray ionisation (CSI) source operated in positive ion mode with a capillary voltage of 1400 V, 3 L/min dry gas and 200 °C dry temperature [60]. For each sample, a data dependent MS/MS spectral acquisition (DDA) in a parallel accumulation-serial fragmentation (PASEF) mode was performed [61].

#### Data analysis and visualisation

Results of the DDA experiments were converted to Mascot generic file format (∗ mgf) and searched by MASCOT (version 2.6, Matrix Science, England) against the BM metagenome. The parameters for the protein database search are presented in Additional file 8 (Table S13.1). Subsequently, mgf files and dat files were loaded into the MetaProteomeAnalyzer (MPA) software (version 3.0) released in 2015 [62]. Three different types of search engines were used for peptide spectral matching: X!Tandem [63], OMSSA [64] and MASCOT (version 2.6, Matrix Science, London, England) [65]. General parameter settings of MPA are shown in Additional file 2 (Table S13.2). A false discovery rate of 1% was used for all samples. The results of MPA search were exported for the peptides and assigned to the BM metagenome database. The aligned metaproteins results were further imported in the excel file for further analysis. Results were visualised using MATLAB (version R2020b; Mathworks, Inc.). PAST4.03 software was used for statistical analyses of PCA [66]. For details: see Additional file 5 and 7. To exclude intracellular enzymes (proteases, peptidases and GH and GT) of microorganisms that degrade biomass, the location of proteins (extra-, intracellular or transmembrane) was determined utilizing the online tool SignalP 6.0 Server (https://services.healthtech.dtu.dk/service.php?SignalP) [67] (for details: see the Additional file 10 and 11). Using KEGG database webpage and the KEGG mapper tool (https://www.genome.jp/kegg/tool/map_pathway3.html), the pathways of the central carbon and energy metabolism were reconstructed for the core community members of the BM process (for details: see Additional file 6 and 9. Significant changes in means of selected samples in BM-24/0 and BM-12/12 are colour coded (i.e., *t* test BM-24/0 and BM-12/12; *p* value < 0.05).

## Supplementary Information

**Additional file 1.** Contains the results of MPA software for the correlation of metagenome and metaproteome results with FDR 1%. Data were normalised based on the total spectral count and the results of the pivot analysis presented in Fig. 2C of the main manuscript.

**Additional file 2: Table S2.1.** Metagenome assembly and binning results. **Table S2.2.** Taxonomic affiliation of metagenome-assembled genomes (MAGs) based on GDTB results. **Table S2.3.** Number of metagenome sequences mapped onto the MAG genome, relative abundance, metagenome completeness and contamination of the 12 identified MAGs.

**Additional file 3: Figure S3.1.** Sodium dodecyl sulphate–polyacrylamide gel electrophoresis (SDS–PAGE) using 25 µg protein extract of the sample of the corresponding H_2_-feeding scheme. (A) Protein profile of four samples of BM-24/0. (B) Protein profile of five samples of BM-12/12. (C) Protein profiles of three samples of BM-18/6 and BM-6/18. (D) Protein profiles of five samples of BM-12/12–20%.

**Additional file 4.** Generation of PCA data presented in Fig. 3B of the main manuscript.

**Additional file 5.** It includes variation in identified proteins of each MAG for BM-24/0 and BM-12/12 using the Kyoto encyclopaedia of genes and genomes (KEGG) map of the central carbon metabolism (map01200). The colours correspond to logarithmic expression ratio [X = Log_2_ (KO _BM-12/12_/KO _BM-24/0_)]. p > 0.05 considered significant, p ≤ 0.05 see colour legend at the right side of each map (*t* test). KO (KEGG Orthology) values are the mean normalised values of the abundance of defined orthologs in the metaproteins of the BM pattern. Each of the KEGG map reported protein variations in the one MAG as presented in these figures: **Figure S5.1.** *Methanobacterium* (MAG5), **Figure S5.2.** *Methanobacterium* (MAG3), **Figure S5.3.** *Methanobacteriaceae* (MAG14), **Figure S5.4.** *Methanobacteriaceae* (MAG8), **Figure S5.5.** *Petrimonas mucosa* (MAG16), **Figure S5.6.** *Sporomusa sphaeroides* (MAG13), **Figure S5.7.** *Defluviitoga tunisiensis* (MAG6), **Figure S5.8.** *Limnochordia* (MAG7), **Fig. S5.9.** *Lutispora* (MAG12), **Figure S5.10.** *Firmicutes* (MAG9), **Figure S5.11.** *Firmicutes* (MAG15), **Figure S5.12.** *Bacteriodales* (MAG10), **Figur S5.13.** *Bacteriodales*(MAG11).

**Additional file 6.** PCA data generation for Fig. 4

**Additional file 7: Table S7.1.** Hydrogenase enzymes of *Methanothrix* during discontinuous H_2_-feeding experiments. The numbers represent the spectral count of the annotated metaproteins F_420_-dependent hydrogenase (Frh) and viologen-reducing hydrogenase (Vht); the number in brackets corresponds to the different proteins identified.

**Additional file 8:** Data generation for illustration of the Kyoto encyclopaedia of genes and genomes (KEGG) pathway of *Methanothrix* in Fig. 5 of the main manuscript.

**Additional file 9:** Data generated for calculation of the relative abundance of extracellular proteases and peptidases (Fig. 6A) of the main manuscript.

**Additional file 10:** Data generated for calculation of the relative abundance of extracellular glycoside hydrolases and glycosyltransferase (Fig. 6B) of the main manuscript.

**Additional file 11:** Includes the spectral counts for metaproteins assigned to the viruses, phages, CRISPR and antimicrobials.

**Additional file 12: Figure S12.1.** The Kyoto encyclopaedia of genes and genomes (KEGG) map of the central carbon metabolism (map01200) of *Methanothrix* in the biological methanation reactor based on metagenomic data.

**Additional file 13: Table S13.1.** Includes preparation of the inoculum and start-up of the biogas reactor. **Table S13.1.** Composition of the applied (1x)  nutrient solution. For the preparation of the nutrient solution, a sterilised filtration unit was used; before use, the solution was degassed with N_2_ for 5 min.

**Additional file 14: Table S14.1.** Parameters setting of the Mascot Deamon software, **Table S14.2.** Parameter settings of the MetaproteomeAnalyzer (MPA) software.

**Additional file 15:** Includes calculations for H_2_ production amount based on mole and litter in the polymer electrolyte membrane water electrolysis process based on Faraday’s law.

**Additional file 16: Table S16.1.** Discontinuous H_2_-feeding experiment sample names in data processing and their correspondence name in PRIDE are presented below. For data analysis, the results of ten segregated data files of each sample were merged in one file.

## Data Availability

Sequence data sets were deposited in the sequence read archive (SRA) under the Bioproject accession numbers PRJNA846548. All mass spectrometry results were made publicly available by an upload to PRIDE [68], which could be accessed with the accession number PXD034618 (Reviewer Account: Username: reviewer_pxd034618@ebi.ac.uk; Password: Yq2heqO6). See details of the microbial community samples in Additional file 16: Table S16.1.

## Abbreviations

Abs: Absolute
BAM: Binary alignment/map
BM: Biological methanation
CSTR: Continuous stirred tank reactor
CRISPR: Clustered regularly interspaced short palindromic repeats
dat: Data (file extension name)
DDA: Data dependent MS/MS spectral acquisition
DIET: Direct interspecies electron transfer
Ech: Energy conversion hydrogenase
Frh: F_420_-dependent hydrogenase
GC: Gas chromatographic system
GH: Glycosyl hydrolase
GT: Glycosyl transferase
GTDB: Genome taxonomy database
HPLC: High performance liquid chromatography
LC: Liquid chromatography
KEGG: Kyoto encyclopaedia of genes and genomes
KO: KEGG Orthology
MAG: Metagenome-assembled genome
mgf: Mascot generic format (file extension name)
MPA: MetaProteomeAnalyzer
MPR: Methane production rate
MR: Methanation reactor
MS: Tandem mass spectrometer/spectrometry
NCBI: National center for biotechnology information
ORP: Oxidation reduction potential
PASEF: Parallel accumulation-serial fragmentation
PCA: Principal component analysis
PEM: Polymer electrolyte membrane
rel: Relative
RHP: Reductive hexulose phosphate
RuBisCO: Ribulose-1,5-bisphosphat-carboxylase/-oxygenase
SDS–PAGE: Sodium dodecyl sulphate–polyacrylamide gel electrophorese
SAM: Sequence alignment/map
Vht: Viologen-reducing hydrogenase
VRERs: Variable renewable electricity resources
