# Supplementary material for: Adaptation of a microbial community to demand-oriented biological methanation
Source: Biotechnol Biofuels Bioprod. 2022 Nov 16;15:125. doi: 10.1186/s13068-022-02207-w (PMC9670408; doi:10.1186/s13068-022-02207-w)
Supplement: Supplementary file 2 — Additional file 2: Table S2.1. Metagenome assembly and binning results. Table S2.2. Taxonomic affiliation of metagenome-assembled genomes (MAGs) based on GDTB results. Table S2.3. Number of metagenome sequences mapped onto the MAG genome, relative abundance, metagenome completeness and contamination of the 12 identified MAGs. [file 13068_2022_2207_MOESM2_ESM.docx]

**Additional file 2**

*Tab. S2.1 Metagenome assembly and binning results*

| Sample | Metagenome data obtained (Mb) | Assembly size | Number of contigs | Approx. N50 | Approx. N90 | Number of genes^1^ | MAGs ^2^ |
| --- | --- | --- | --- | --- | --- | --- | --- |
| [BM-Fermenter](https://emgb.cebitec.uni-bielefeld.de/magdeburg/emgb/) | 2,874 | 53,834,983 | 16,735 | 9,000 | 105,200 | 61,685 | 12 |

^1^ Including genes being classified as "partial" applying program Prodigal 2.6.3.

^2^ MAGs with completeness above 80%, and contamination rate below 10%.

*Tab. S2.2* *Taxonomic affiliation of metagenome-assembled genomes (MAGs) based on GDTB results.*

|  | **Metagenome- assembled  genome  (MAG)** | **Taxonomic affiliation  (based on GDTB results)** | | | | | | |
| --- | --- | --- | --- | --- | --- | --- | --- | --- |
|  |  | **Superkingdom** | **Phylum** | **Class** | **Order** | **Family** | **Genus** | **Species** |
|  | 2 | *Archaea* | *Euryarchaeota* | *Methanosarcinia* | *Methanotrichales* | *Methanotrichaceae* | *Methanothrix* | *Unknown* |
|  | 3 | *Archaea* | *Euryarchaeota* | *Methanobacteria* | *Methanobacteriales* | *Methanobacteriaceae* | *Methanobacterium* | *Unknown* |
|  | 5 | *Archaea* | *Euryarchaeota* | *Methanobacteria* | *Methanobacteriales* | *Methanobacteriaceae* | *Methanobacterium* | *Unknown* |
|  | 6 | *Bacteria* | *Thermotogota* | *Thermotogota* | *Petrotogales* | *Petrotogaceae* | *Defluviitoga* | *Defluviitoga*  *tunisiensis* |
|  | 7 | *Bacteria* | *Firmicutes* | *Limnochordia* | *Unknown* | *Unknown* | *Unknown* | *Unknown* |
|  | 9 | *Bacteria* | *Firmicutes* | *Unknown* | *Unknown* | *Unknown* | *Unknown* | *Unknown* |
|  | 10 | *Bacteria* | *Bacteroidetes* | *Bacteroidia* | *Bacteroidales* | *Unknown* | *Unknown* | *Unknown* |
|  | 11 | *Bacteria* | *Bacteroidetes* | *Bacteroidia* | *Bacteroidales* | *Unknown* | *Unknown* | *Unknown* |
|  | 13 | *Bacteria* | *Firmicutes* | *Negativicutes* | *Sporomusales* | *Sporomusaceae* | *Sporomusa* | *Sporomusa*  *sphaeroides* |
|  | 14 | *Archaea* | *Euryarchaeota* | *Methanobacteria* | *Methanobacteriales* | *Methanobacteriaceae* | *Unknown* | *Unknown* |
|  | 15 | *Bacteria* | *Firmicutes* | *Unknown* | *Unknown* | *Unknown* | *Unknown* | *Unknown* |
|  | 16 | *Bacteria* | *Bacteroidetes* | *Bacteroidia* | *Bacteroidales* | *Dysgomonadaceae* | *Petrimonas* | *Petrimonas*  *mucosa* |
|  |  |  |  |  |  |  |  |  |

*Tab. S2.3 N*umber of metagenome sequences mapped onto the MAG genome, relative abundance*, metagenome completeness and contamination of the 12 identified MAGs .*

|  |  |  |  |  |
| --- | --- | --- | --- | --- |
| **Metagenome-assembled  genome (MAG)** | **Completeness [%]** | **Contamination* [%]** | **Number of metagenome sequences mapped onto the MAG genome** | **Relative abundance to the entire metagenome (%) **** |
| 2 | 97.7 | 0.0 | 513,547 | 6.68 |
| 3 | 98.4 | 0.0 | 151,497 | 1.97 |
| 5 | 95.7 | 1.1 | 1,259,005 | 16.39 |
| 6 | 98.3 | 0.9 | 91,554 | 1.19 |
| 7 | 95.1 | 2.3 | 81,497 | 1.06 |
| 9 | 94.3 | 3.4 | 77,466 | 1.00 |
| 10 | 93.0 | 0.5 | 181,800 | 2.36 |
| 11 | 90.0 | 1.7 | 82,135 | 1.06 |
| 13 | 90.8 | 4.3 | 120,51 | 0.15 |
| 14 | 80.0 | 0.0 | 1,383,101 | 18.01 |
| 15 | 95.1 | 3.3 | 668,591 | 8.70 |
| 16 | 98.4 | 0.6 | 194,841 | 2.53 |

* No single copy marker genes detected.

** The total number of raw reads after downstream processing: 7,678,940
