# Supplementary material for: Adaptation of a microbial community to demand-oriented biological methanation
Source: Biotechnol Biofuels Bioprod. 2022 Nov 16;15:125. doi: 10.1186/s13068-022-02207-w (PMC9670408; doi:10.1186/s13068-022-02207-w)
Supplement: Supplementary file 3 — Additional file 3: Figure S3.1. Sodium dodecyl sulphate–polyacrylamide gel electrophoresis (SDS–PAGE) using 25 µg protein extract of the sample of the corresponding H2-feeding scheme. (A) Protein profile of four samples of BM-24/0. (B) Protein profile of five samples of BM-12/12. (C) Protein profiles of three samples of BM-18/6 and BM-6/18. (D) Protein profiles of five samples of BM-12/12–20%. [file 13068_2022_2207_MOESM3_ESM.docx]

**Additional file 3**


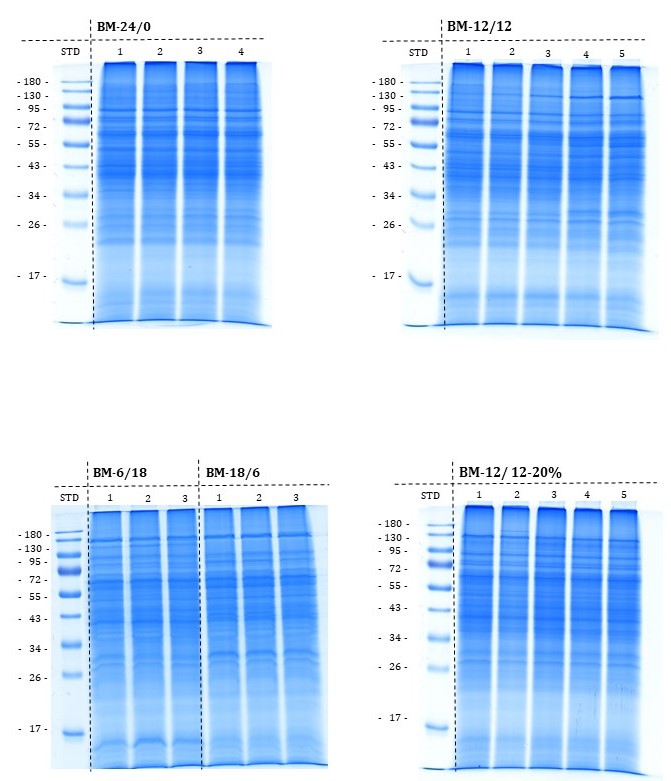


(D)

(C)

(B)

(A)

*Fig. S3.1 Sodium dodecyl sulphate polyacrylamide gel electrophoresis (SDS–PAGE) of different H_2_-feedings experiments using 25 µg protein extract of the corresponding feeding scheme. (A) Protein profile of four samples of BM-24/0. (B) Protein profile of five samples of BM-12/12. (C) Protein profiles of three samples of BM-18/6 and BM-6/18. (D) Protein profiles of five samples of BM-12/12-20%.*
