# Supplementary material for: Adaptation of a microbial community to demand-oriented biological methanation
Source: Biotechnol Biofuels Bioprod. 2022 Nov 16;15:125. doi: 10.1186/s13068-022-02207-w (PMC9670408; doi:10.1186/s13068-022-02207-w)
Supplement: Supplementary file 5 — Additional file 5. It includes variation in identified proteins of each MAG for BM-24/0 and BM-12/12 using the Kyoto encyclopaedia of genes and genomes (KEGG) map of the central carbon metabolism (map01200). The colours correspond to logarithmic expression ratio [X = Log2 (KO BM-12/12/KO BM-24/0)]. p > 0.05 considered significant, p ≤ 0.05 see colour legend at the right side of each map (t test). KO (KEGG Orthology) values are the mean normalised values of the abundance of defined orthologs in the metaproteins of the BM pattern. Each of the KEGG map reported protein variations in the one MAG as presented in these figures: Figure S5.1. Methanobacterium (MAG5), Figure S5.2. Methanobacterium (MAG3), Figure S5.3. Methanobacteriaceae (MAG14), Figure S5.4. Methanobacteriaceae (MAG8), Figure S5.5. Petrimonas mucosa (MAG16), Figure S5.6. Sporomusa sphaeroides (MAG13), Figure S5.7. Defluviitoga tunisiensis (MAG6), Figure S5.8. Limnochordia (MAG7), Fig. S5.9. Lutispora (MAG12), Figure S5.10. Firmicutes (MAG9), Figure S5.11. Firmicutes (MAG15), Figure S5.12. Bacteriodales (MAG10), Figur S5.13. Bacteriodales(MAG11). [file 13068_2022_2207_MOESM5_ESM.docx]

**Additional file 5**

***Central carbon metabolism of Methanobacterium (MAG5)***

*
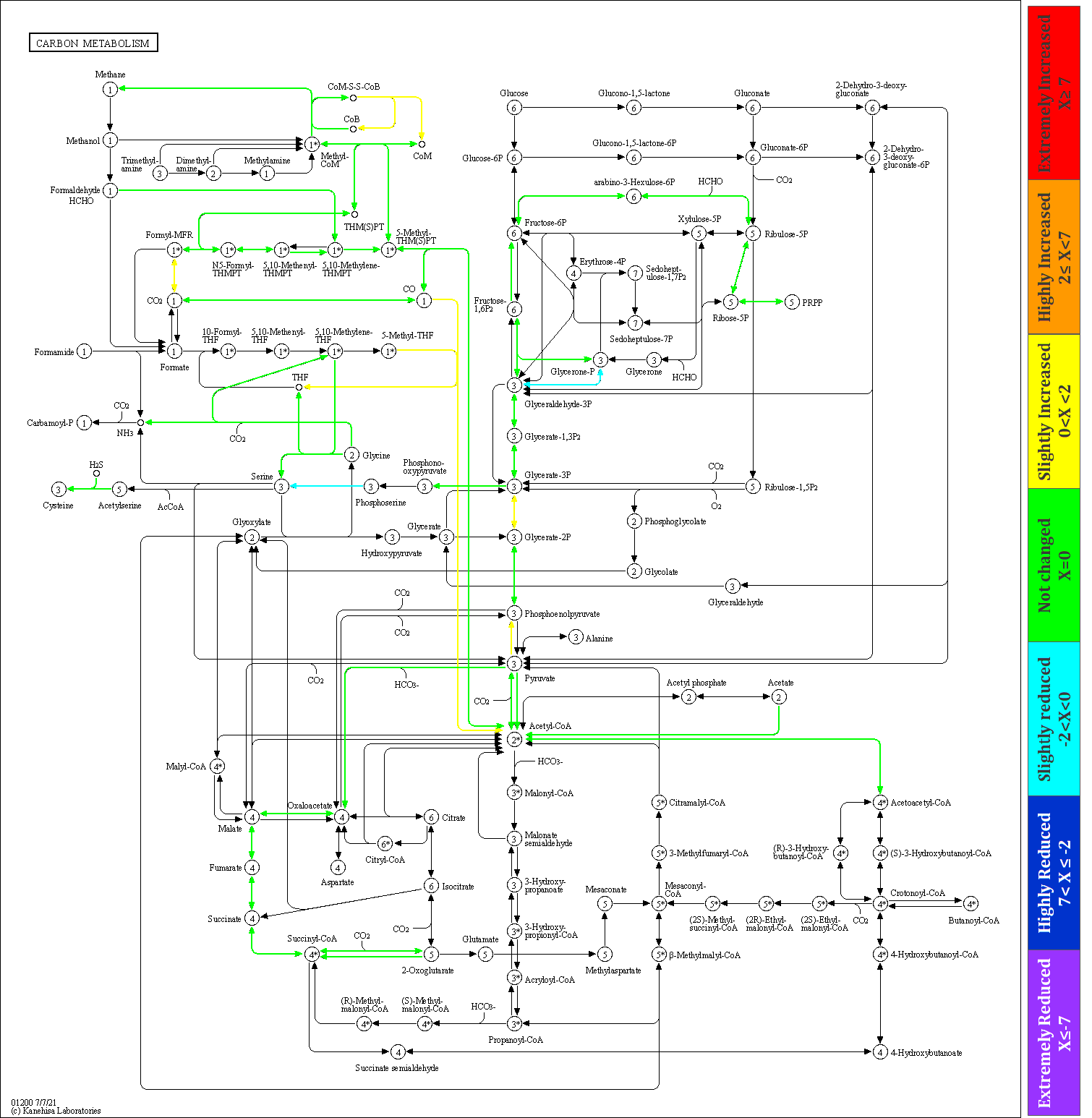
*

Fig. S5.1 Variation in identified Methanobacterium (MAG5) proteins for BM-24/0 and BM-12/12 using the Kyoto encyclopaedia of genes and genomes (KEGG) map of the central carbon metabolism (map01200). The colours correspond to logarithmic expression ratio [X=Log_2_ (KO _BM-12/12_/KO _BM-24/0_)]. p>0.05 considered significant, p<0.05 see colour legend at the right (t-test). KO (KEGG Orthology) values are the mean normalised values of the abundance of defined orthologs in the metaproteins of the BM pattern.

***Central carbon metabolism of Methanobacterium (MAG3)***

*
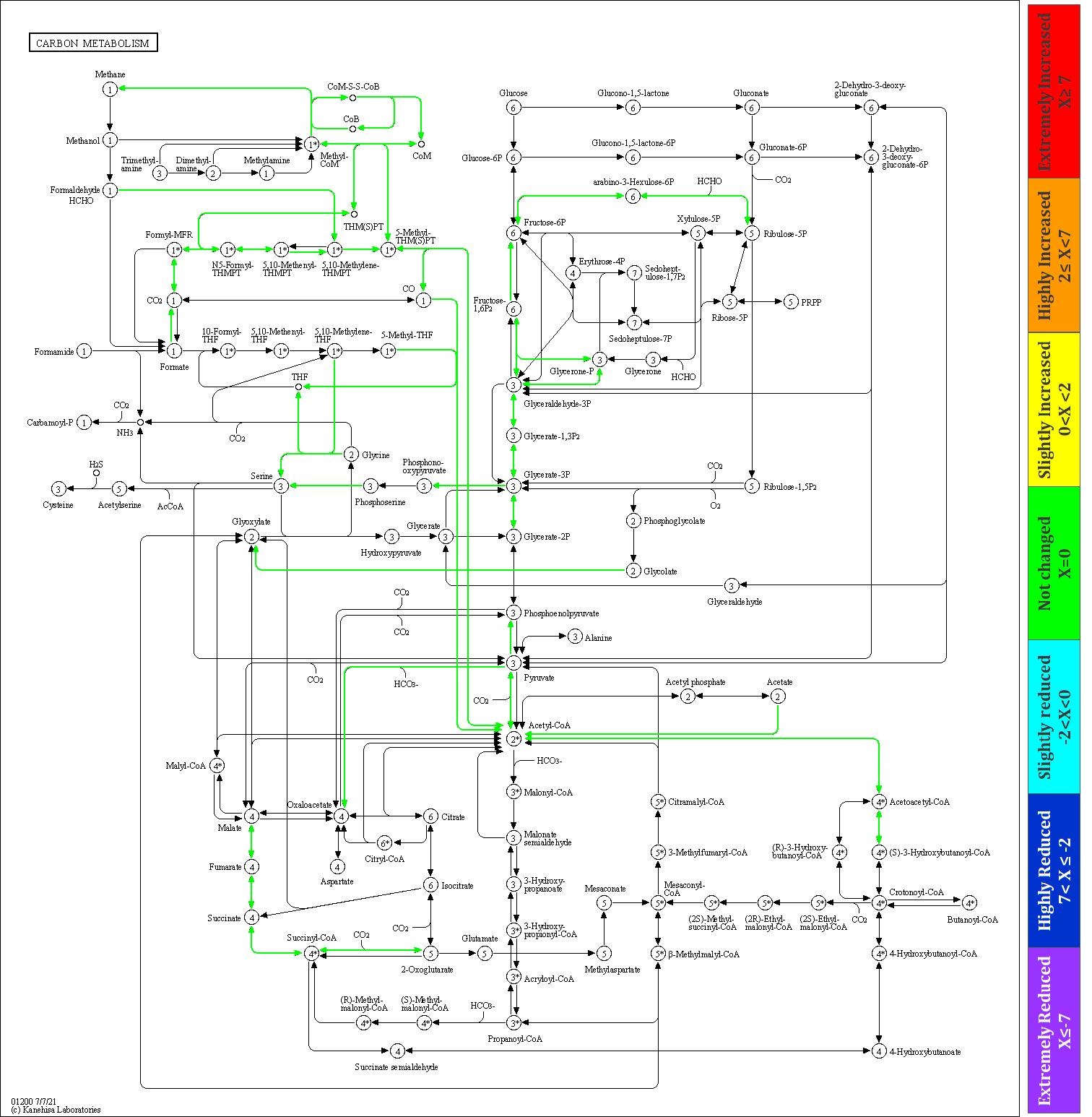
*

Fig. S5.2 Variation in identified Methanobacterium (MAG3) proteins for BM-24/0 and BM-12/12 using the Kyoto encyclopaedia of genes and genomes (KEGG) map of the central carbon metabolism (map01200). The colours correspond to logarithmic expression ratio [X=Log_2_ (KO _BM-12/12_/KO _BM-24/0_)]. p>0.05 considered significant, p<0.05 see colour legend at the right (t-test). KO (KEGG Orthology) values are the mean normalised values of the abundance of defined orthologs in the metaproteins of the BM pattern.

***Central carbon metabolism of Methanobacteriaceae (MAG14)***

*
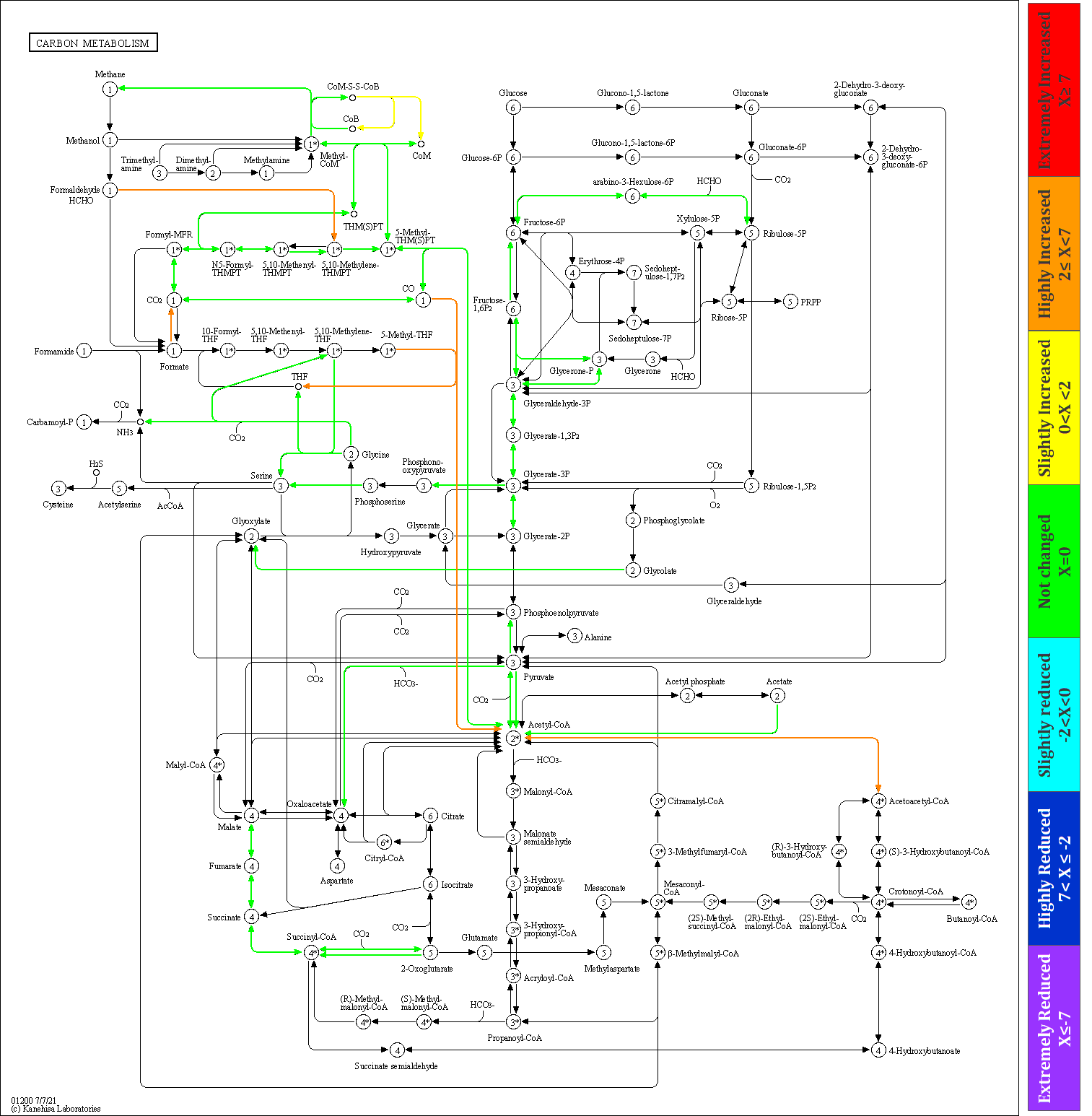
*

Fig. S5.3 Variation in identified Methanobacteriaceae (MAG14) proteins for BM-24/0 and BM-12/12 using the Kyoto encyclopaedia of genes and genomes (KEGG) map of the central carbon metabolism (map01200). The colours correspond to logarithmic expression ratio [X=Log_2_ (KO _BM-12/12_/KO _BM-24/0_)]. p>0.05 considered significant, p<0.05 see colour legend at the right (t-test). KO (KEGG Orthology) values are the mean normalised values of the abundance of defined orthologs in the metaproteins of the BM pattern.

***Central carbon metabolism of Methanobacteriaceae (MAG8)***

*
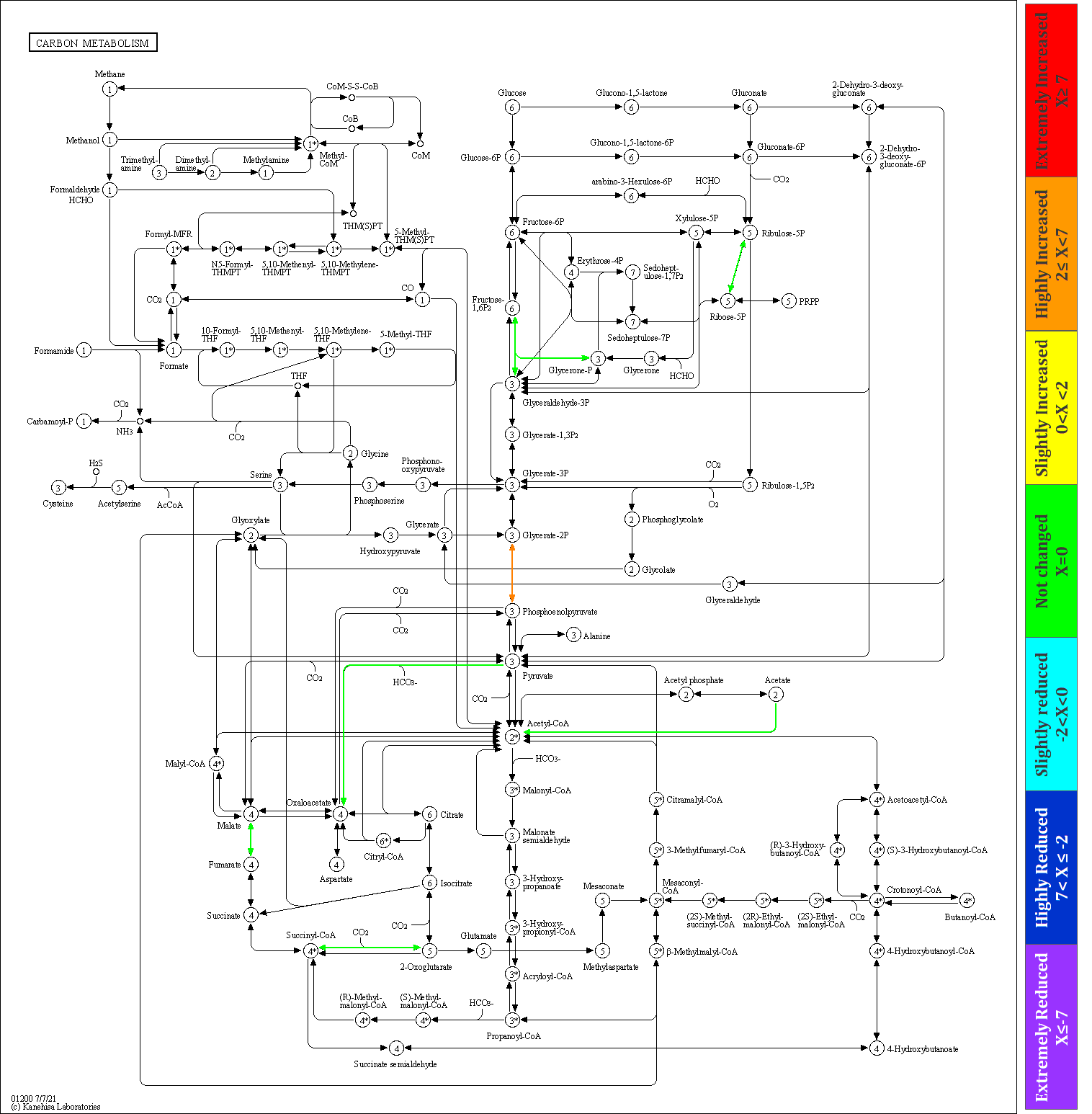
*

Fig. S5.4 Variation in identified Methanobacteriaceae (MAG12) proteins for BM-24/0 and BM-12/12 using the Kyoto encyclopaedia of genes and genomes (KEGG) map of the central carbon metabolism (map01200). The colours correspond to logarithmic expression ratio [X=Log_2_ (KO _BM-12/12_/KO _BM-24/0_)]. p>0.05 considered significant, p<0.05 see colour legend at the right (t-test). KO (KEGG Orthology) values are the mean normalised values of the abundance of defined orthologs in the metaproteins of the BM pattern.

***Central carbon metabolism of Petrimonas mucosa***

*
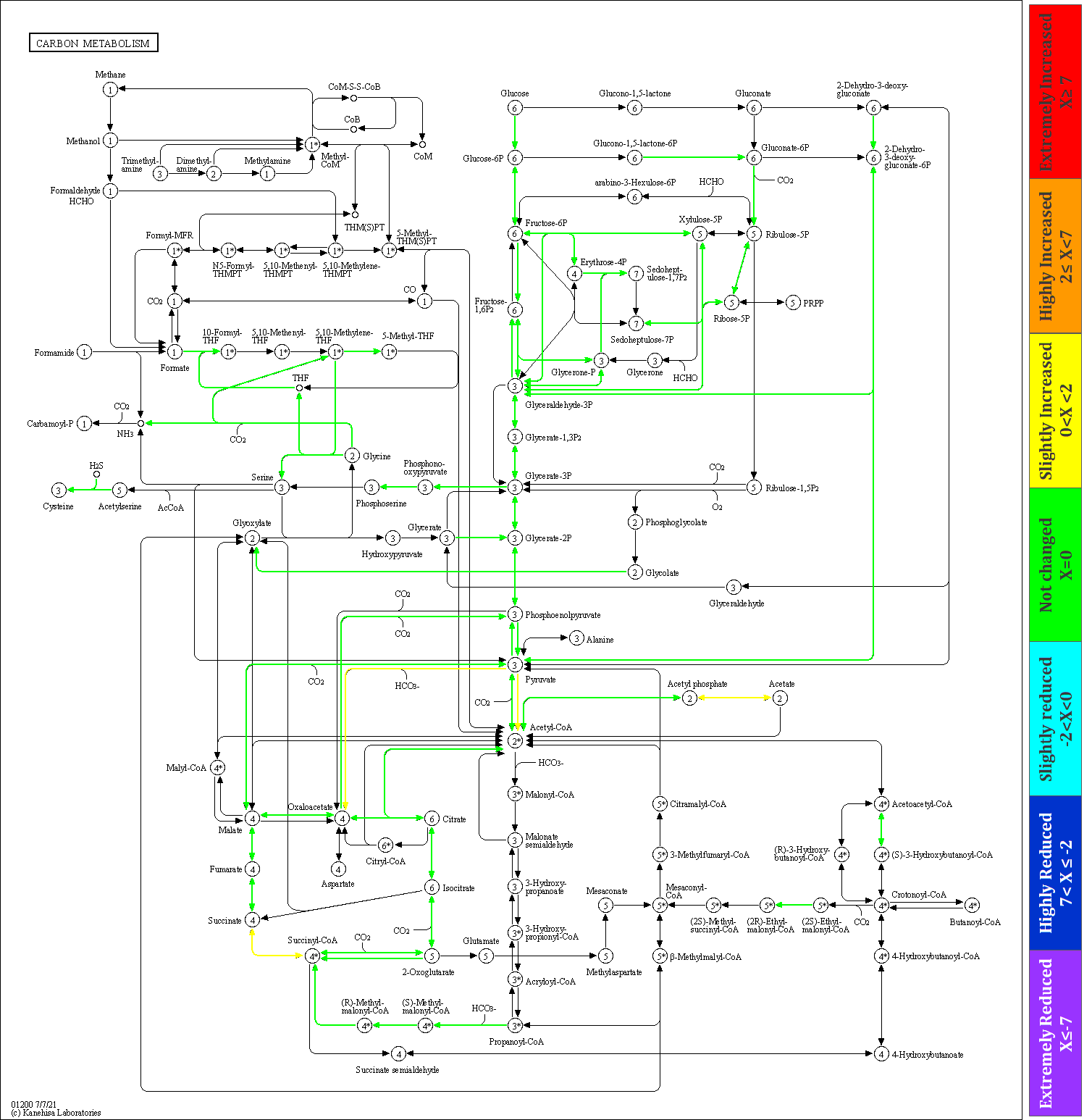
*

Fig. S5.5 Variation in identified Petrimonas mucosa proteins for BM-24/0 and BM-12/12 using the Kyoto encyclopaedia of genes and genomes (KEGG) map of the central carbon metabolism (map01200). The colours correspond to logarithmic expression ratio [X=Log_2_ (KO _BM-12/12_/KO _BM-24/0_)]. p>0.05 considered significant, p<0.05 see colour legend at the right (t-test). KO (KEGG Orthology) values are the mean normalised values of the abundance of defined orthologs in the metaproteins of the BM pattern.

***Central carbon metabolism of Sporomusa sphaeroides***

*
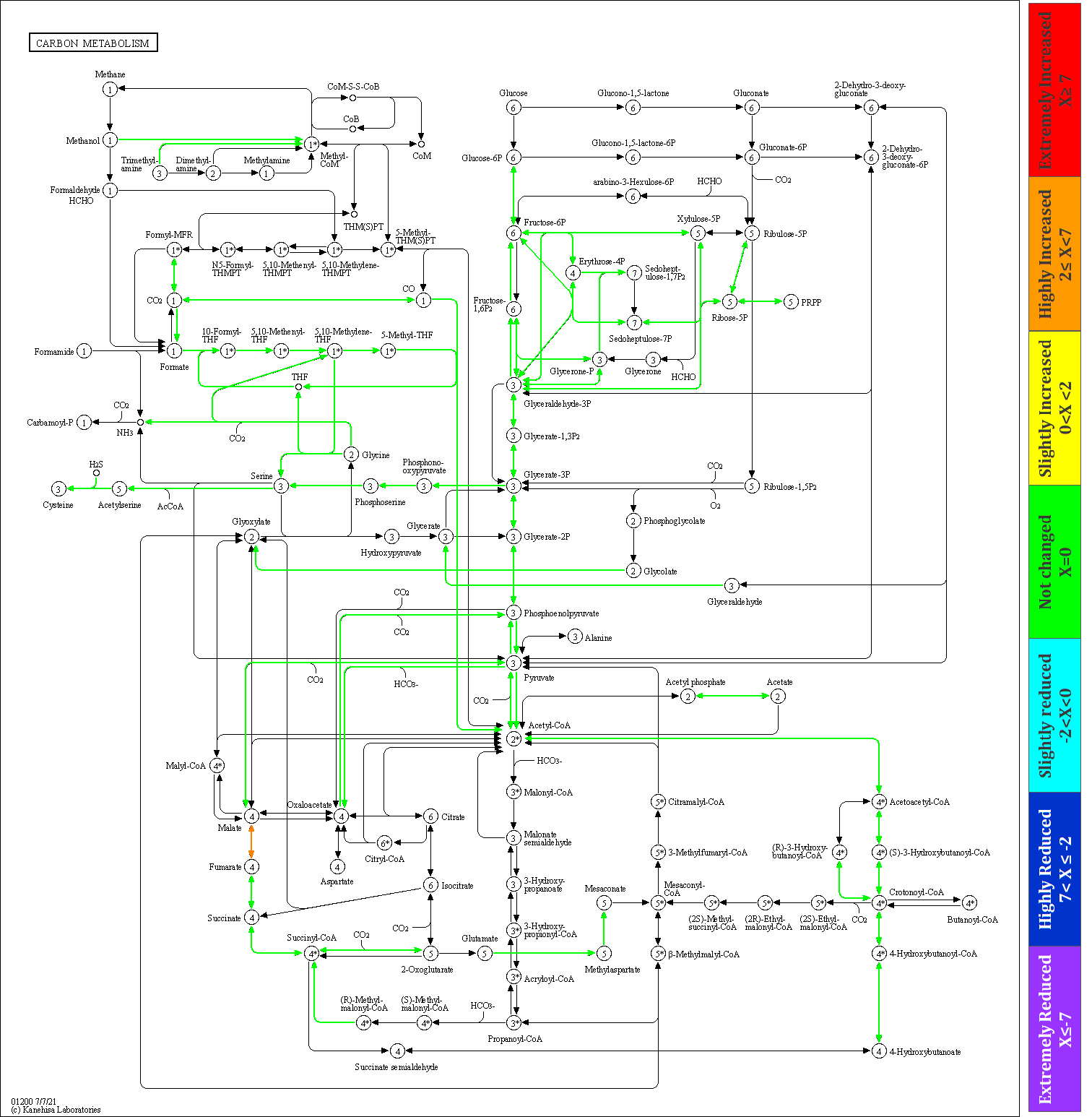
*

Fig. S5.6 Variation in identified Sporomusa sphaeroides proteins for BM-24/0 and BM-12/12 using the Kyoto encyclopaedia of genes and genomes (KEGG) map of the central carbon metabolism (map01200). The colours correspond to logarithmic expression ratio [X=Log_2_ (KO _BM-12/12_/KO _BM-24/0_)]. p>0.05 considered significant, p<0.05 see colour legend at the right (t-test). KO (KEGG Orthology) values are the mean normalised values of the abundance of defined orthologs in the metaproteins of the BM pattern.

***Central carbon metabolism of Defluviitoga tunisiensis***

*
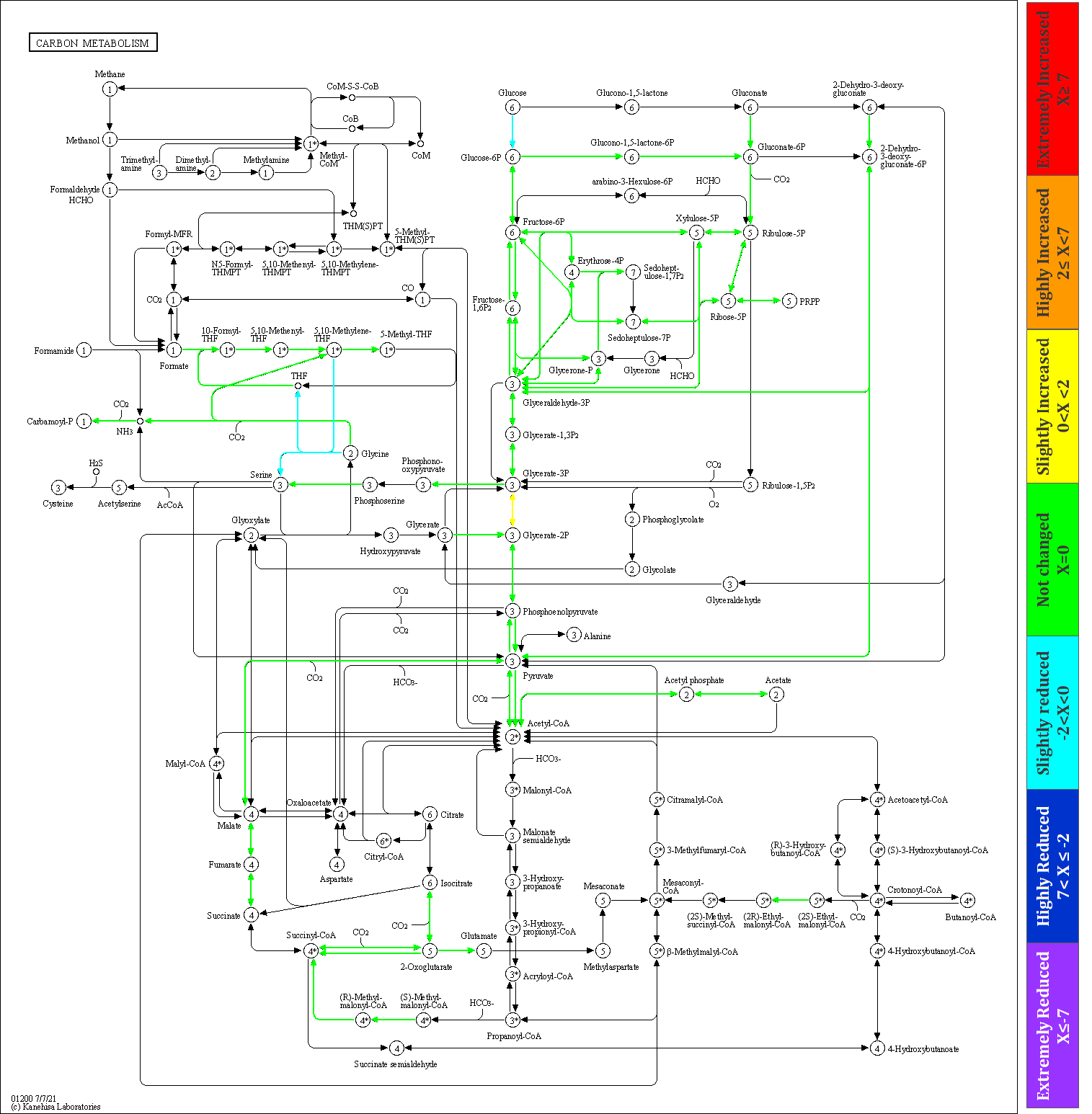
*

Fig. S5.7 Variation in identified Defluviitoga tunisiensis proteins for BM-24/0 and BM-12/12 using the Kyoto encyclopaedia of genes and genomes (KEGG) map of the central carbon metabolism (map01200). The colours correspond to logarithmic expression ratio [X=Log_2_ (KO _BM-12/12_/KO _BM-24/0_)]. p>0.05 considered significant, p<0.05 see colour legend at the right (t-test). KO (KEGG Orthology) values are the mean normalised values of the abundance of defined orthologs in the metaproteins of the BM pattern.

***Central carbon metabolism of Limnochordia***

*
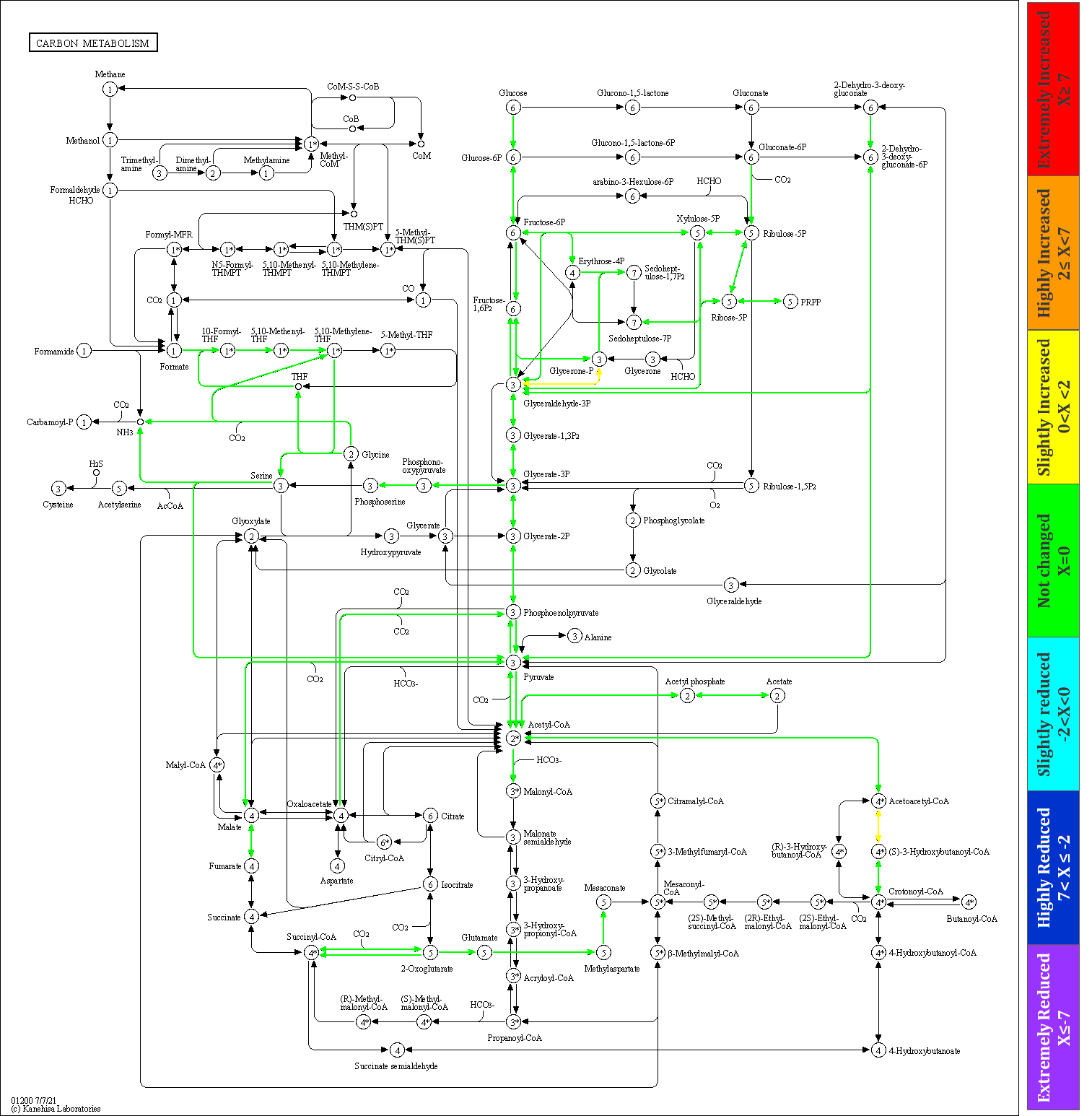
*

Fig. S5.8 Variation in identified Limnochordia proteins for BM-24/0 and BM-12/12 using the Kyoto encyclopaedia of genes and genomes (KEGG) map of the central carbon metabolism (map01200). The colours correspond to logarithmic expression ratio [X=Log_2_ (KO _BM-12/12_/KO _BM-24/0_)]. p>0.05 considered significant, p<0.05 see colour legend at the right (t-test). KO (KEGG Orthology) values are the mean normalised values of the abundance of defined orthologs in the metaproteins of the BM pattern.

***Central carbon metabolism of Lutispora***

*
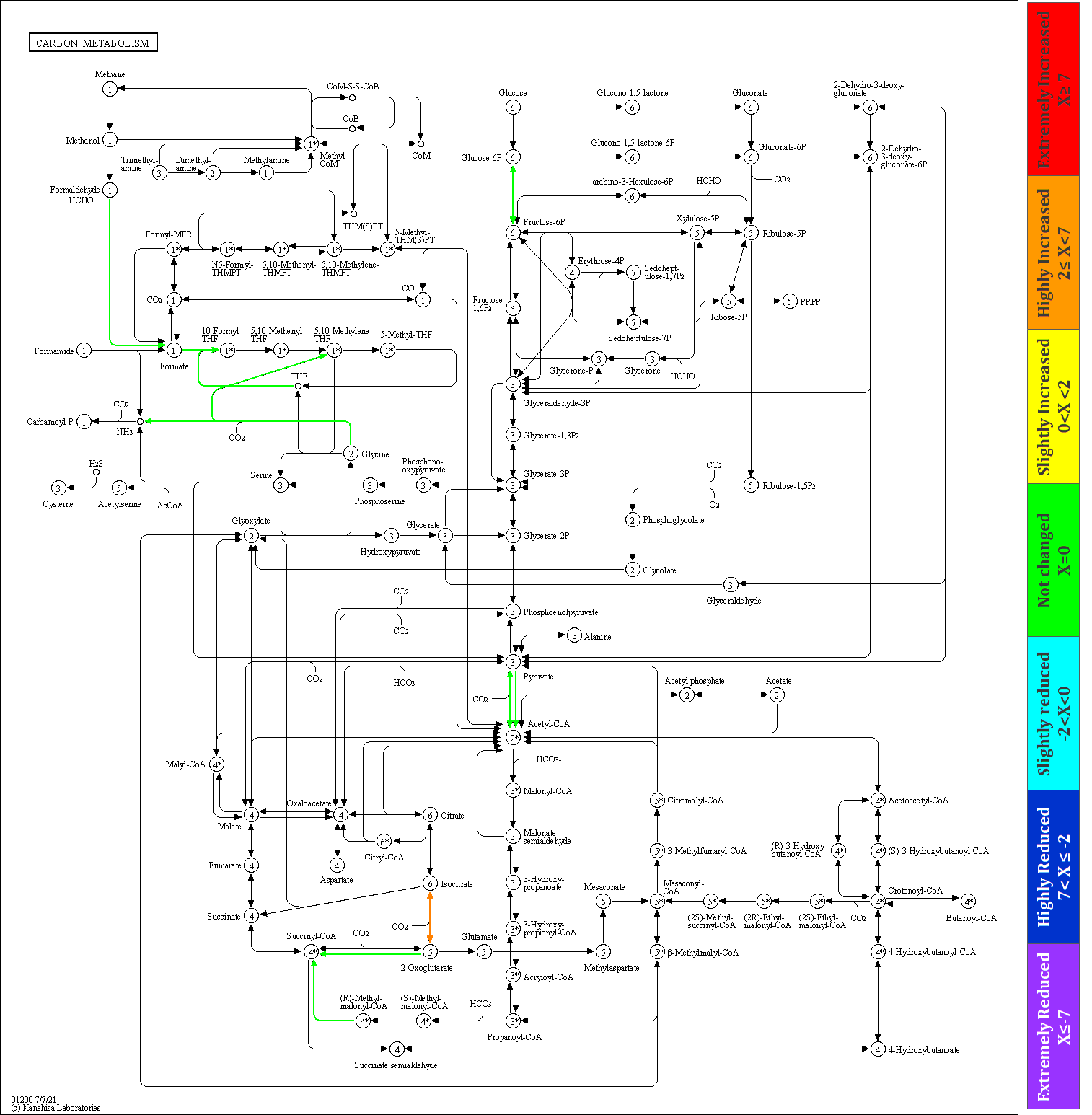
*

Fig. S5.9 Variation in identified Lutispora proteins for BM-24/0 and BM-12/12 using the Kyoto encyclopaedia of genes and genomes (KEGG) map of the central carbon metabolism (map01200). The colours correspond to logarithmic expression ratio [X=Log_2_ (KO _BM-12/12_/KO _BM-24/0_)]. p>0.05 considered significant, p<0.05 see colour legend at the right (t-test). KO (KEGG Orthology) values are the mean normalised values of the abundance of defined orthologs in the metaproteins of the BM pattern.

***Central carbon metabolism of Firmicutes (MAG9)***

*
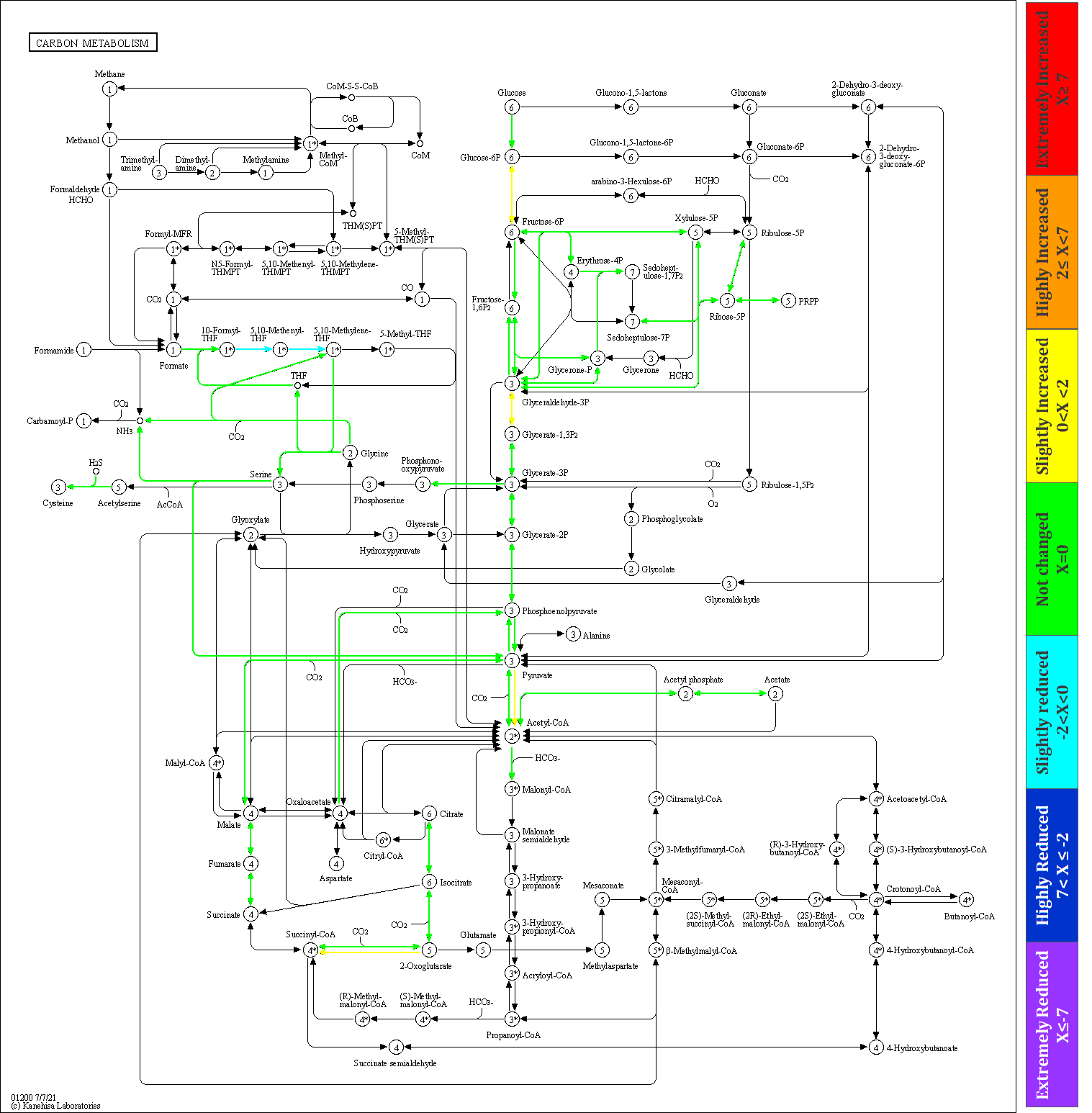
*

Fig. S5.10 Variation in identified Firmicutes (MAG9) proteins for BM-24/0 and BM-12/12 using the Kyoto encyclopaedia of genes and genomes (KEGG) map of the central carbon metabolism (map01200). The colours correspond to logarithmic expression ratio [X=Log_2_ (KO _BM-12/12_/KO _BM-24/0_)]. p>0.05 considered significant, p<0.05 see colour legend at the right (t-test). KO (KEGG Orthology) values are the mean normalised values of the abundance of defined orthologs in the metaproteins of the BM pattern.

***Central carbon metabolism of Firmicutes (MAG15)***

*
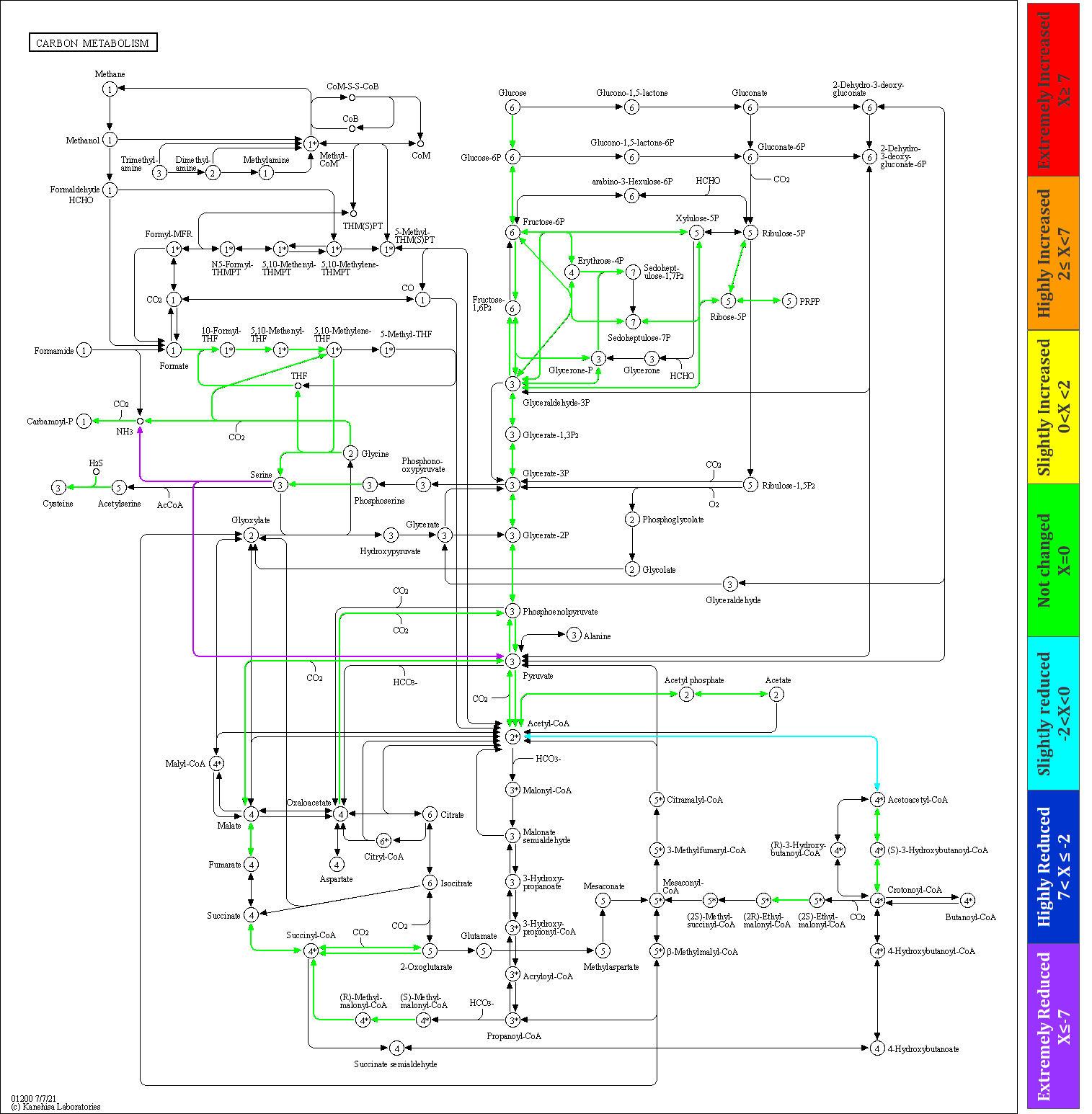
*

Fig. S5.11 Variation in identified Firmicutes (MAG15) proteins for BM-24/0 and BM-12/12 using the Kyoto encyclopaedia of genes and genomes (KEGG) map of the central carbon metabolism (map01200). The colours correspond to logarithmic expression ratio [X=Log_2_ (KO _BM-12/12_/KO _BM-24/0_)]. p>0.05 considered significant, p<0.05 see colour legend at the right (t-test). KO (KEGG Orthology) values are the mean normalised values of the abundance of defined orthologs in the metaproteins of the BM pattern.

***Central carbon metabolism of Bacteroidales (MAG10)***

*
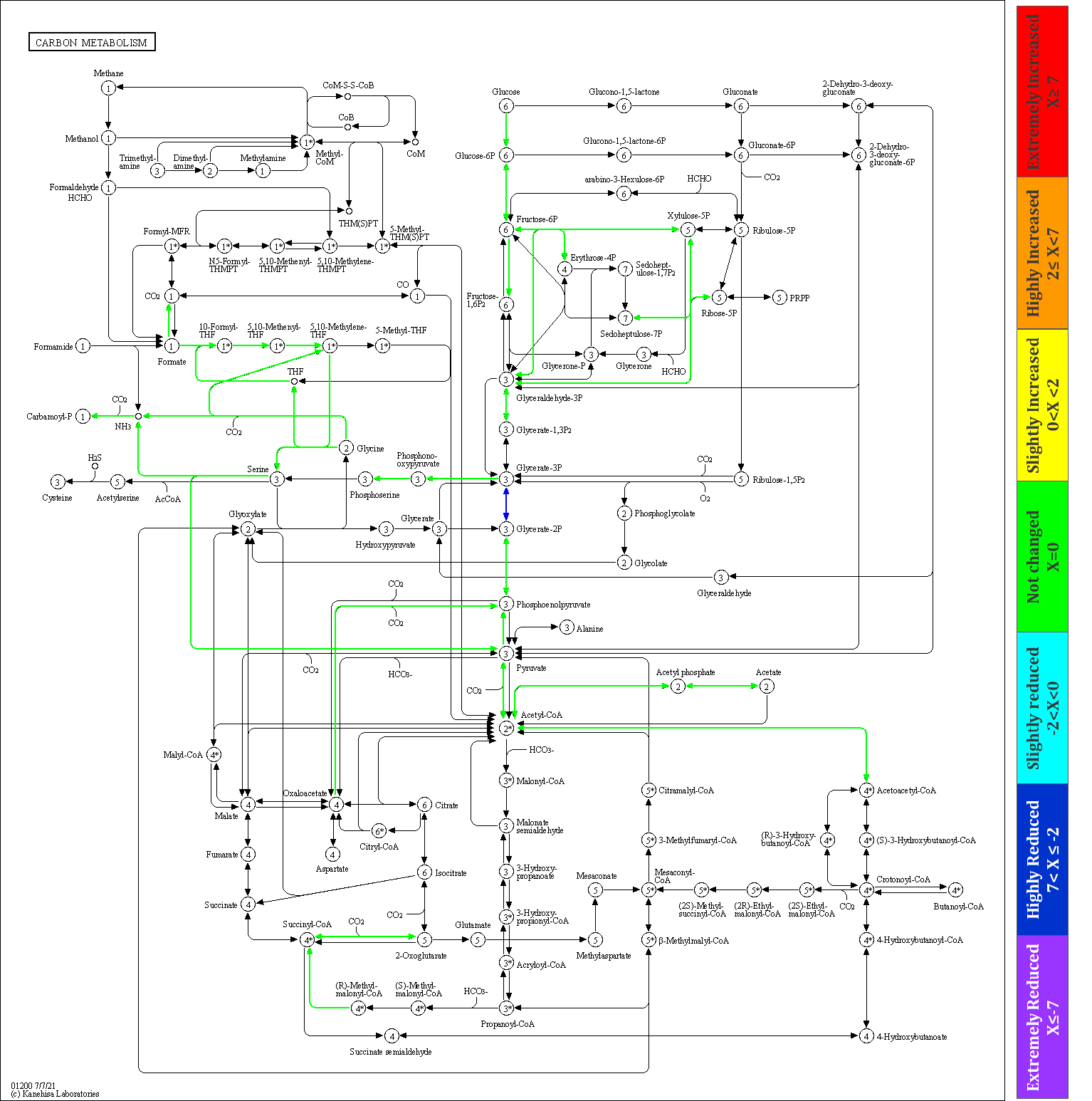
*

Fig. S5.12 Variation in identified Bacteroidales (MAG10) proteins for BM-24/0 and BM-12/12 using the Kyoto encyclopaedia of genes and genomes (KEGG) map of the central carbon metabolism (map01200). The colours correspond to logarithmic expression ratio [X=Log_2_ (KO _BM-12/12_/KO _BM-24/0_)]. p>0.05 considered significant, p<0.05 see colour legend at the right (t-test). KO (KEGG Orthology) values are the mean normalised values of the abundance of defined orthologs in the metaproteins of the BM pattern.

***Central carbon metabolism of Bacteroidales (MAG11)***

*
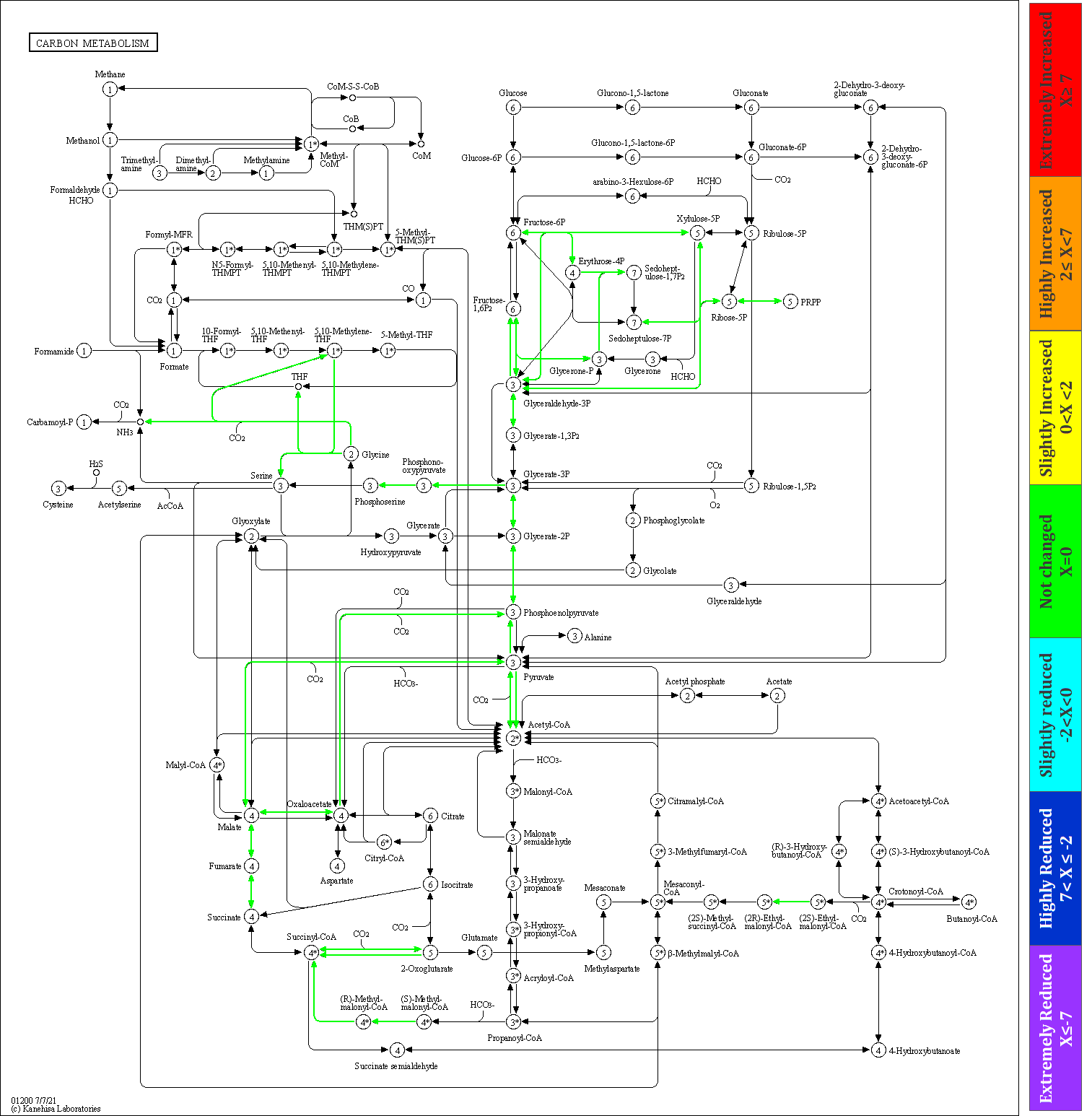
*

Fig. S5.13 Variation in identified Bacteroidales (MAG11) proteins for BM-24/0 and BM-12/12 using the Kyoto encyclopaedia of genes and genomes (KEGG) map of the central carbon metabolism (map01200). The colours correspond to logarithmic expression ratio [X=Log_2_ (KO _BM-12/12_/KO _BM-24/0_)]. p>0.05 considered significant, p<0.05 see colour legend at the right (t-test). KO (KEGG Orthology) values are the mean normalised values of the abundance of defined orthologs in the metaproteins of the BM pattern.
