# Supplementary material for: Adaptation of a microbial community to demand-oriented biological methanation
Source: Biotechnol Biofuels Bioprod. 2022 Nov 16;15:125. doi: 10.1186/s13068-022-02207-w (PMC9670408; doi:10.1186/s13068-022-02207-w)
Supplement: Supplementary file 7 — Additional file 7: Table S7.1. Hydrogenase enzymes of Methanothrix during discontinuous H2-feeding experiments. The numbers represent the spectral count of the annotated metaproteins F420-dependent hydrogenase (Frh) and viologen-reducing hydrogenase (Vht); the number in brackets corresponds to the different proteins identified. [file 13068_2022_2207_MOESM7_ESM.docx]

**Additional file 7**

Tab. S7.1 Hydrogenase enzymes of Methanothrix during discontinuous H_2_-feeding experiments. The numbers represent the spectral count of the annotated metaproteins F_420_-dependent hydrogenase (Frh) and viologen-reducing hydrogenase (Vht); the number in brackets corresponds to the different proteins identified.
