# Supplementary material for: Adaptation of a microbial community to demand-oriented biological methanation
Source: Biotechnol Biofuels Bioprod. 2022 Nov 16;15:125. doi: 10.1186/s13068-022-02207-w (PMC9670408; doi:10.1186/s13068-022-02207-w)
Supplement: Supplementary file 12 — Additional file 12: Figure S12.1. The Kyoto encyclopaedia of genes and genomes (KEGG) map of the central carbon metabolism (map01200) of Methanothrix in the biological methanation reactor based on metagenomic data. [file 13068_2022_2207_MOESM12_ESM.docx]

**Additional file 12**


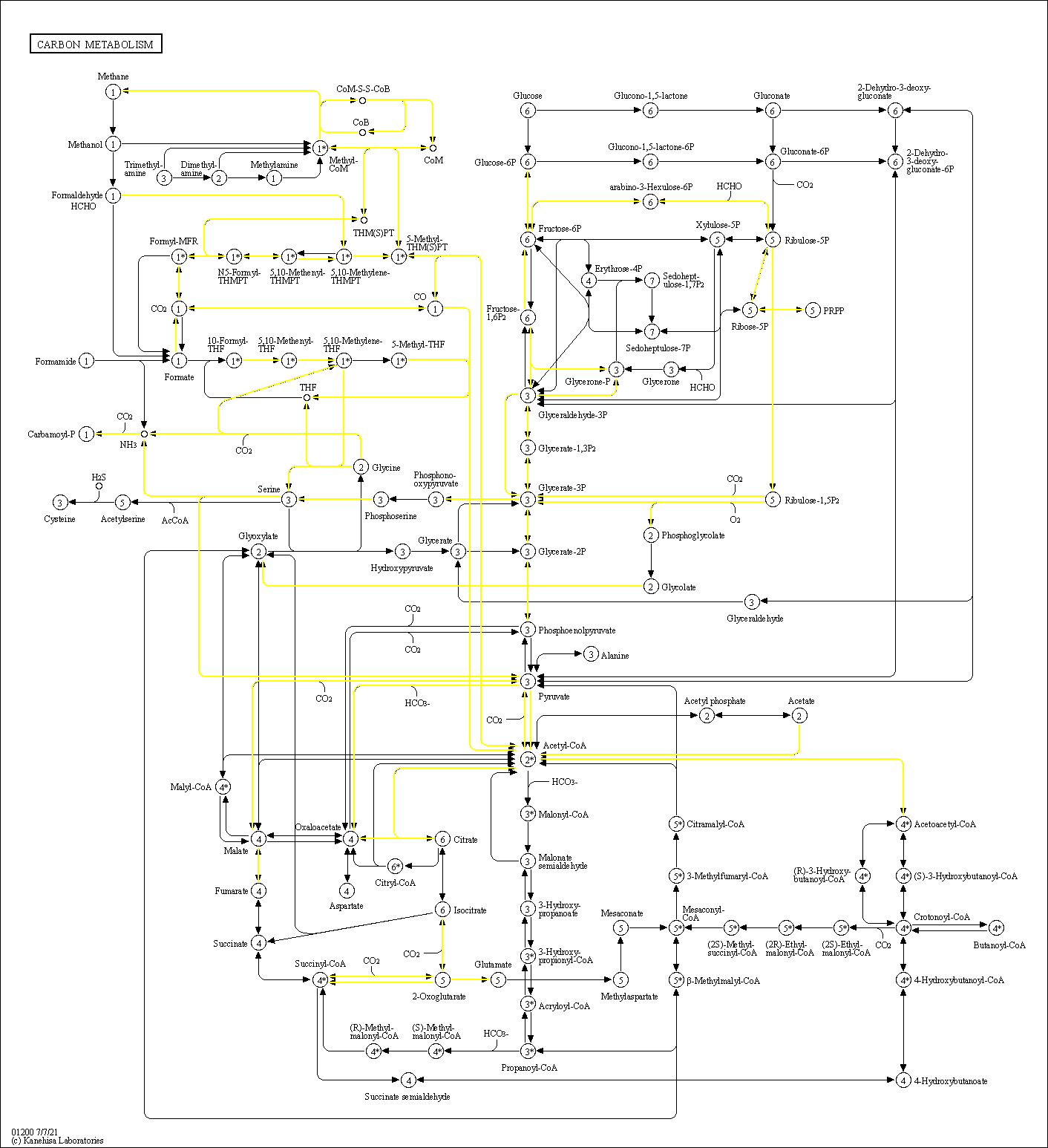


*Fig. S12.1. The Kyoto encyclopaedia of genes and genomes (KEGG) map of the central carbon metabolism (map01200) of Methanothrix in the biological methanation reactor based on metagenomic data.*
