# Supplementary material for: Adaptation of a microbial community to demand-oriented biological methanation
Source: Biotechnol Biofuels Bioprod. 2022 Nov 16;15:125. doi: 10.1186/s13068-022-02207-w (PMC9670408; doi:10.1186/s13068-022-02207-w)
Supplement: Supplementary file 13 — Additional file 13: Table S13.1. Includes preparation of the inoculum and start-up of the biogas reactor. Table S13.1. Composition of the applied (1x) nutrient solution. For the preparation of the nutrient solution, a sterilised filtration unit was used; before use, the solution was degassed with N2 for 5 min. [file 13068_2022_2207_MOESM13_ESM.docx]

**Additional file 13**

**S13-1 Preparation of the inoculum and start-up of the biogas reactor**

As inoculum, fresh biomass (5 L) of a local BGP located near Magdeburg (Germany) was used. For homogenisation prior to use, about 200 mL of the supernatant of the sludge (centrifuged at 3000$\times$g, 50 mL vessels, 3 min at room temperature). After flushing of the reactor (500 mL working volume, Sixfors multi bioreactor system, INFORS AG, Bottmingen, Switzerland) with N_2_ gas, the supernatant was used for primary inoculation (40 °C, stirring at 100 rpm by a magnetic propeller stirrer (INFORS AG, Bottmingen, Switzerland)). A second inoculation with 200 mL was done the next day using the same procedure. In the first two months, the reactor was stepwise harvested and fed by hand to keep the pH between 7-8 and the volume constant. The nutrient solution is given in Table 1; the glucose concentration was 11.5 (g/L). After adaptation of the culture to the lowest feeding rate (20 mL/d), the pumps of the Sixfors multi bioreactor system were used for pulse feeding at the rate of 20 mL/d. The exhaust biogas was cooled with a gas condenser supplied with 20°C cold water to remove any aqueous vapour from the produced biogas. Biogas production and composition were controlled by an “GärOnA” gas analysis system (GärOnA, Gesellschaft zur Förderung von Medizin-, Bio- und Umwelttechnologien e. V. (GMBU), Halle, Germany; mobilGC Elektrochemie Halle GmbH (ECH), Halle, Germany). The enriched biomass obtained was used to inoculate the biogas and biological methanation reactors as described in the experimental setup and operation conditions of the main manuscript.

Tab. S13.1: Composition of the applied 1x nutrient solution. For the preparation of the nutrient solution, a sterilised filtration unit was used; before use, the solution was degassed with N_2_ for 5 min.

| **Nutrient** | **Molecular Formula** | **Final Concentration (g/L)** |
| --- | --- | --- |
| Glucose | C_6_H_12_O_6_ | 1.15E+01 |
| Urea | CH_4_N_2_O | 2.56E+00 |
| Cysteine hydrochloride monohydrate | C_3_H_7_NO_2_S ∙ HCl ∙ H_2_O | 5.00E-01 |
| Calcium chloride dihydrate | CaCl_2_ ∙ 2H_2_O | 5.30E-02 |
| Dipotassium hydrogen phosphate | K_2_HPO_4_ | 2.20E+00 |
| Magnesium chloride hexahydrate | MgCl_2_ ∙ 6H_2_O | 5.06E-01 |
| Iron (II) sulphate heptahydrate | FeSO_4_ ∙ 7H_2_O | 5.12E-02 |
| Sodium chloride | NaCl | 7.00E-03 |
| Cobalt chloride hexahydrate | CoCl_2_ ∙ 6H_2_O | 1.00E-02 |
| Copper (II) chloride dihydrate | CuCl_2_ ∙ 2H_2_O | 5.00E-04 |
| Boric acid | H_3_BO_3_ | 1.50E-02 |
| Manganese (II) chloride thetrahydratetetrahydrate | MnCl_2_ ∙ 4H_2_O | 1.50E-03 |
| Sodium molybdate dihydrate | Na_2_MoO_4_ ∙ 2H_2_O | 1.50E-03 |
| Sodium selenite | Na_2_SeO_3_ | 1.00E-03 |
| Nickel (II) chloride hexahydrate | NiCl_2_ ∙ 6H_2_O | 1.00E-03 |
| Zinc sulphate heptahydrate | ZnSO_4_ ∙ 7H_2_O | 3.60E-01 |
| Lipoic acid | C_8_H_14_O_2_S_2_ | 5.00E-06 |
| Pyridoxine hydrochloride | C_8_H_11_NO_3_ ∙ HCl | 1.00E-05 |
| Thiamine hydrochloride dihydrate | C_12_H_17_ClN_4_OS ∙ HCl ∙ 2H_2_O | 5.00E-06 |
| Riboflavin | C_17_H_20_N_4_O_6_ | 5.00E-06 |
| Vitamin B12 | C₆₃H₈₈CoN₁₄O₁₄P | 1.00E-07 |
| Nicotinic acid | C_6_H_5_NO_2_ | 5.00E-06 |
| D-Calcium-pantothenate | C_18_H_32_CaN_2_O_10_ | 5.00E-06 |
| Biotin | C_10_H_16_N_2_O_3_S | 2.00E-06 |
| Folic acid | C_19_H_19_N_7_O_6_ | 2.00E-06 |
| p-Aminobenzoic acid | C_7_H_7_NO_2_ | 5.00E-06 |
