# Supplementary material for: Adaptation of a microbial community to demand-oriented biological methanation
Source: Biotechnol Biofuels Bioprod. 2022 Nov 16;15:125. doi: 10.1186/s13068-022-02207-w (PMC9670408; doi:10.1186/s13068-022-02207-w)
Supplement: Supplementary file 14 — Additional file 14: Table S14.1. Parameters setting of the Mascot Deamon software, Table S14.2. Parameter settings of the MetaproteomeAnalyzer (MPA) software. [file 13068_2022_2207_MOESM14_ESM.docx]

**Additional file 14**

Tab. S14.1: Parameter settings of the Mascot Deamon software

| **Parameter** | **Setting** |
| --- | --- |
| Data Bank | Biological methanation metagenome (BM1)  (SRA accession number: SRR19568451) |
| Fixed modifications | Carbamidomethyl (cysteine) |
| Variable modification | Oxidation (methionine) |
| Enzyme | Trypsin |
| Missed cleavage (max) | 1 |
| Peptide charge | +2, +3, +4 |
| Peptide tolerance | 0.02 Da |
| MS/MS tolerance | 0.02 Da |
| FDR | 1 |
| Search strategy | Decoy |
| Mass | Monoisotopic |
| Data Format | Mascot generic |
| Instrument type | ESI-QUAD-TOF |

| **Parameter** | **Setting** |
| --- | --- |
| Data Bank | Biological methanation metagenome (BM1)  (SRA accession number: SRR19568451) |
| Search engines | X!Tandem, OMSSA, Mascot |
| Precursor ion tolerance | 0.02 Da |
| Fragment ion tolerance | 0.02 Da |
| Missed cleavage (max) | 1 |
| Search strategy | Target-decoy |
| Transfer | 1000 spectra per package |

Tab. S14.2: Parameter settings of the MetaproteomeAnalyzer (MPA) software
