# Supplementary material for: Adaptation of a microbial community to demand-oriented biological methanation
Source: Biotechnol Biofuels Bioprod. 2022 Nov 16;15:125. doi: 10.1186/s13068-022-02207-w (PMC9670408; doi:10.1186/s13068-022-02207-w)
Supplement: Supplementary file 15 — Additional file 15: Includes calculations for H2 production amount based on mole and litter in the polymer electrolyte membrane water electrolysis process based on Faraday’s law. [file 13068_2022_2207_MOESM15_ESM.docx]

**Additional file 15**

Assuming the ideal gas law, hydrogen production of the polymer electrolyte membrane water electrolyser was calculated based on the second Faraday law as follows:

$Q=I\times t \left( A\times s \right)$(S15-1)

$Q=n\times z\times F \left( A\times s \right)$(S15-2)

Where Q is the electric charge ((A$\times$s) or C), I is the current (A), t is the time (s), n is the amount of substance (in this case H_2_ (mole)), z is the number of electrons required for separating a molecule of substance (z(H_2_) =2) and F is Faraday constant (96485 (A$\times$s)/mol).

The amount of H_2_ produced in 1 (s) and by applying 1 (A) ( $\varphi_{\mathrm{mole}}$) is calculated as follows:

$\varphi_{\mathrm{mole}}=\frac{n}{I\times t}=\frac{1}{z\times F}=\frac{1}{2\times96485 \frac{(A\times S)}{\mathrm{mol}}}=5.1812\times{10}^{-6} (\frac{\mathrm{mol}}{\left( A\times s \right)})$ (S15-3)

Considering the molar volume of gas at standard conditions is 22.414 (L),$\varphi_{\mathrm{vol}}$ or the volume of H_2_ produced in 1 (min) by applying 1 (A) is calculated as follows:

$\varphi_{\mathrm{vol}}=\varphi_{\mathrm{mole}}\times22.414\left( \frac{\mathrm{NL}}{\mathrm{mole}} \right)\times60 (\frac{s}{\min}) = 6.696\times{10}^{-3}\left( \frac{\mathrm{NL}}{\left( A\times\min\right)} \right)$ (S15-4)
