# Supplementary material for: Adaptation of a microbial community to demand-oriented biological methanation
Source: Biotechnol Biofuels Bioprod. 2022 Nov 16;15:125. doi: 10.1186/s13068-022-02207-w (PMC9670408; doi:10.1186/s13068-022-02207-w)
Supplement: Supplementary file 16 — Additional file 16: Table S16.1. Discontinuous H2-feeding experiment sample names in data processing and their correspondence name in PRIDE are presented below. For data analysis, the results of ten segregated data files of each sample were merged in one file. [file 13068_2022_2207_MOESM16_ESM.docx]

**Additional file 16**

*Tab. S16.1 Discontinuous H_2_-feeding experiment sample names in data processing and their correspondence name in PRIDE are presented below. For data analysis, the results of ten segregated data files of each sample were merged in one file.*

| Sample Name | PRIDE Name | Sample Name | PRIDE Name |
| --- | --- | --- | --- |
| BM-24/0-1 | BM-24-0-1_1_RA1 | **BM-24/0-2** | BM-24-0-2_1_RB3 |
|  | BM-24-0-1_2_RA2 |  | BM-24-0-2_2_RB4 |
|  | BM-24-0-1_3_RA3 |  | BM-24-0-2_3_RB5 |
|  | BM-24-0-1_4_RA4 |  | BM-24-0-2_4_RB6 |
|  | BM-24-0-1_5_RA5 |  | BM-24-0-2_5_RB7 |
|  | BM-24-0-1_6_RA6 |  | BM-24-0-2_6_RB8 |
|  | BM-24-0-1_7_RA7 |  | BM-24-0-2_7_RC1 |
|  | BM-24-0-1_8_RA8 |  | BM-24-0-2_8_RC2 |
|  | BM-24-0-1_9_RB1 |  | BM-24-0-2_9_RC3 |
|  | BM-24-0-1_10_RB2 |  | BM-24-0-2_10_RC4 |
| BM-24/0-3 | BM-24-0-3_1_RC5 | **BM-24/0-4** | BM-24-0-4_1_GA1 |
|  | BM-24-0-3_2_RC6 |  | BM-24-0-4_2_GA2 |
|  | BM-24-0-3_3_RC7 |  | BM-24-0-4_3_GA3 |
|  | BM-24-0-3_4_RC8 |  | BM-24-0-4_4_GA4 |
|  | BM-24-0-3_5_RD1 |  | BM-24-0-4_5_GA5 |
|  | BM-24-0-3_6_RD2 |  | BM-24-0-4_6_GA6 |
|  | BM-24-0-3_7_RD3 |  | BM-24-0-4_7_GA7 |
|  | BM-24-0-3_8_RD4 |  | BM-24-0-4_8_GA8 |
|  | BM-24-0-3_9_RD5 |  | BM-24-0-4_9_GB1 |
|  | BM-24-0-3_10_RD6 |  | BM-24-0-4_10_GB2 |
| BM-12/12-1 | BM-12-12-1_1_GB3 | **BM-12/12-2** | BM-12-12-2_1_GA1 |
|  | BM-12-12-1_2_GB4 |  | BM-12-12-2_2_GA2 |
|  | BM-12-12-1_3_GB5 |  | BM-12-12-2_3_GA3 |
|  | BM-12-12-1_4_GB6 |  | BM-12-12-2_4_GA4 |
|  | BM-12-12-1_5_GB7 |  | BM-12-12-2_5_GA5 |
|  | BM-12-12-1_6_GB8 |  | BM-12-12-2_6_GA6 |
|  | BM-12-12-1_7_GC1 |  | BM-12-12-2_7_GA7 |
|  | BM-12-12-1_8_GC2 |  | BM-12-12-2_8_GA8 |
|  | BM-12-12-1_9_GC3 |  | BM-12-12-2_9_GB1 |
|  | BM-12-12-1_10_GC4 |  | BM-12-12-2_10_GB2 |
| BM-12/12-3 | BM-12-12-3_1_GB3 | **BM-12/12-4** | BM-12-12-4_1_GC5 |
|  | BM-12-12-3_2_GB4 |  | BM-12-12-4_2_GC6 |
|  | BM-12-12-3_3_GB5 |  | BM-12-12-4_3_GC7 |
|  | BM-12-12-3_4_GB6 |  | BM-12-12-4_4_GC8 |
|  | BM-12-12-3_5_GB7 |  | BM-12-12-4_5_GD1 |
|  | BM-12-12-3_6_GB8 |  | BM-12-12-4_6_GD2 |
|  | BM-12-12-3_7_GC1 |  | BM-12-12-4_7_GD3 |
|  | BM-12-12-3_8_GC2 |  | BM-12-12-4_8_GD4 |
|  | BM-12-12-3_9_GC3 |  | BM-12-12-4_9_GD5 |
|  | BM-12-12-3_10_GC4 |  | BM-12-12-4_10_GD6 |
| BM-12/12-3 | BM-12-12-5_1_GA1 | **BM-6/18-1** | BM-18-6-1_1_GD7 |
|  | BM-12-12-5_2_GA2 |  | BM-18-6-1_2_GD8 |
|  | BM-12-12-5_3_GA3 |  | BM-18-6-1_3_GE1 |
|  | BM-12-12-5_4_GA4 |  | BM-18-6-1_4_GE2 |
|  | BM-12-12-5_5_GA5 |  | BM-18-6-1_5_GE3 |
|  | BM-12-12-5_6_GA6 |  | BM-18-6-1_6_GE4 |
|  | BM-12-12-5_7_GA7 |  | BM-18-6-1_7_GE5 |
|  | BM-12-12-5_8_GA8 |  | BM-18-6-1_8_GE6 |
|  | BM-12-12-5_9_GB1 |  | BM-18-6-1_9_GE7 |
|  | BM-12-12-5_10_GB2 |  | BM-18-6-1_10_GE8 |
| BM-18/6-2 | BM-18-6-2_1_BA1 | **BM-18/6-3** | BM-18-6-3_1_BD1 |
|  | BM-18-6-2_2_BA2 |  | BM-18-6-3_2_BD2 |
|  | BM-18-6-2_3_BA3 |  | BM-18-6-3_3_BD3 |
|  | BM-18-6-2_4_BA4 |  | BM-18-6-3_4_BD4 |
|  | BM-18-6-2_5_BA5 |  | BM-18-6-3_5_BD5 |
|  | BM-18-6-2_6_BA6 |  | BM-18-6-3_6_BD6 |
|  | BM-18-6-2_7_BA7 |  | BM-18-6-3_7_BD7 |
|  | BM-18-6-2_8_BA8 |  | BM-18-6-3_8_BD8 |
|  | BM-18-6-2_9_BB1 |  | BM-18-6-3_9_BE1 |
|  | BM-18-6-2_10_BB2 |  | BM-18-6-3_10_BE2 |
| BM-6/18-1 | BM-6-18-1_1_GA1 | **BM-6/18-2** | BM-6-18-2_1_GB3 |
|  | BM-6-18-1_2_GA2 |  | BM-6-18-2_2_GB4 |
|  | BM-6-18-1_3_GA3 |  | BM-6-18-2_3_GB5 |
|  | BM-6-18-1_4_GA4 |  | BM-6-18-2_4_GB6 |
|  | BM-6-18-1_5_GA5 |  | BM-6-18-2_5_GB7 |
|  | BM-6-18-1_6_GA6 |  | BM-6-18-2_6_GB8 |
|  | BM-6-18-1_7_GA7 |  | BM-6-18-2_7_GC1 |
|  | BM-6-18-1_8_GA8 |  | BM-6-18-2_8_GC2 |
|  | BM-6-18-1_9_GB1 |  | BM-6-18-2_9_GC3 |
|  | BM-6-18-1_10_GB2 |  | BM-6-18-2_10_GC4 |
| BM-6/18-3 | BM-6-18-3_1_GC5 | **BM-12/12-20%-1** | BM-12-12-20%-1_1_GA1 |
|  | BM-6-18-3_1_GC6 |  | BM-12-12-20%-1_2_GA2 |
|  | BM-6-18-3_1_GC7 |  | BM-12-12-20%-1_3_GA3 |
|  | BM-6-18-3_1_GC8 |  | BM-12-12-20%-1_4_GA4 |
|  | BM-6-18-3_1_GD1 |  | BM-12-12-20%-1_5_GA5 |
|  | BM-6-18-3_1_GD2 |  | BM-12-12-20%-1_6_GA6 |
|  | BM-6-18-3_1_GD3 |  | BM-12-12-20%-1_7_GA7 |
|  | BM-6-18-3_1_GD4 |  | BM-12-12-20%-1_8_GA8 |
|  | BM-6-18-3_1_GD5 |  | BM-12-12-20%-1_9_GB1 |
|  | BM-6-18-3_1_GD6 |  | BM-12-12-20%-1_10_GB2 |
| BM-12/12-20%-2 | BM-12-12-20%-2_1_GB3 | **BM-12/12-20%-3** | BM-12-12-20%-3_1_GC5 |
|  | BM-12-12-20%-2_2_GB4 |  | BM-12-12-20%-3_2_GC6 |
|  | BM-12-12-20%-2_3_GB5 |  | BM-12-12-20%-3_3_GC7 |
|  | BM-12-12-20%-2_4_GB6 |  | BM-12-12-20%-3_4_GC8 |
|  | BM-12-12-20%-2_5_GB7 |  | BM-12-12-20%-3_5_GD1 |
|  | BM-12-12-20%-2_6_GB8 |  | BM-12-12-20%-3_6_GD2 |
|  | BM-12-12-20%-2_7_GC1 |  | BM-12-12-20%-3_7_GD3 |
|  | BM-12-12-20%-2_8_GC2 |  | BM-12-12-20%-3_8_GD4 |
|  | BM-12-12-20%-2_9_GC3 |  | BM-12-12-20%-3_9_GD5 |
|  | BM-12-12-20%-2_10_GC4 |  | BM-12-12-20%-3_10_GD6 |
| BM-12/12-20%-4 | BM-12-12-20%-4_1_GD7 | **BM-12/12-20%-5** | BM-12-12-20%-5_1_GA1 |
|  | BM-12-12-20%-4_2_GD8 |  | BM-12-12-20%-5_2_GA2 |
|  | BM-12-12-20%-4_3_GE1 |  | BM-12-12-20%-5_3_GA3 |
|  | BM-12-12-20%-4_4_GE2 |  | BM-12-12-20%-5_4_GA4 |
|  | BM-12-12-20%-4_5_GE3 |  | BM-12-12-20%-5_5_GA5 |
|  | BM-12-12-20%-4_6_GE4 |  | BM-12-12-20%-5_6_GA6 |
|  | BM-12-12-20%-4_7_GE5 |  | BM-12-12-20%-5_7_GA7 |
|  | BM-12-12-20%-4_8_GE6 |  | BM-12-12-20%-5_8_GA8 |
|  | BM-12-12-20%-4_9_GE7 |  | BM-12-12-20%-5_9_GB1 |
|  | BM-12-12-20%-4_10_GE8 |  | BM-12-12-20%-5_10_GB2 |
